# Progressive Polycomb Assembly on H3K27me3 Compartments Generates Polycomb Bodies with Developmentally Regulated Motion

**DOI:** 10.1371/journal.pgen.1002465

**Published:** 2012-01-19

**Authors:** Thierry Cheutin, Giacomo Cavalli

**Affiliations:** Institut de Génétique Humaine, CNRS UPR 1142, Montpellier, France; Harvard Medical School, Howard Hughes Medical Institute, United States of America

## Abstract

Polycomb group (PcG) proteins are conserved chromatin factors that maintain silencing of key developmental genes outside of their expression domains. Recent genome-wide analyses showed a Polycomb (PC) distribution with binding to discrete PcG response elements (PREs). Within the cell nucleus, PcG proteins localize in structures called PC bodies that contain PcG-silenced genes, and it has been recently shown that PREs form local and long-range spatial networks. Here, we studied the nuclear distribution of two PcG proteins, PC and Polyhomeotic (PH). Thanks to a combination of immunostaining, immuno-FISH, and live imaging of GFP fusion proteins, we could analyze the formation and the mobility of PC bodies during fly embryogenesis as well as compare their behavior to that of the condensed fraction of euchromatin. Immuno-FISH experiments show that PC bodies mainly correspond to 3D structural counterparts of the linear genomic domains identified in genome-wide studies. During early embryogenesis, PC and PH progressively accumulate within PC bodies, which form nuclear structures localized on distinct euchromatin domains containing histone H3 tri-methylated on K27. Time-lapse analysis indicates that two types of motion influence the displacement of PC bodies and chromatin domains containing H2Av-GFP. First, chromatin domains and PC bodies coordinately undergo long-range motions that may correspond to the movement of whole chromosome territories. Second, each PC body and chromatin domain has its own fast and highly constrained motion. In this motion regime, PC bodies move within volumes slightly larger than those of condensed chromatin domains. Moreover, both types of domains move within volumes much smaller than chromosome territories, strongly restricting their possibility of interaction with other nuclear structures. The fast motion of PC bodies and chromatin domains observed during early embryogenesis strongly decreases in late developmental stages, indicating a possible contribution of chromatin dynamics in the maintenance of stable gene silencing.

## Author Summary

The three-dimensional organization of genes and associated proteins is critical for gene regulation. Polycomb group proteins are important developmental regulators controlling the expression of hundreds of genes. They are not homogeneously distributed in the cell nucleus, instead forming nuclear subcompartments called Polycomb bodies. We investigated the dynamics of Polycomb bodies during *Drosophila* embryonic development, demonstrating that two Polycomb proteins, Polycomb and Polyhomeotic, gradually assemble onto bodies enriched in histone H3 trimethylated on lysine 27, a hallmark of Polycomb silencing. Polycomb bodies are not the most condensed euchromatic part of the genome. Instead, a large amount of genomic chromatin is organized in a histone- and DNA–dense structure distinct from Polycomb bodies. Polycomb bodies move, meet, and split dynamically during development. Their motion has two regimes: a fast, highly constrained motion and a slower regime where multiple bodies undergo long-range coordinated movements potentially corresponding to chromosome territory movements. These regimes are not restricted to Polycomb but also extend to bulk “condensed euchromatin,” which is characterized by slower motion and a narrower radius of confinement. Both motion regimes progressively slow down during development, suggesting that regulation of chromatin dynamics may play an important role in the maintenance of gene silencing in differentiated cells.

## Introduction

The biological mechanisms allowing one genome to translate into the many epigenomes that characterize different cell types involve binding of regulatory factors to chromatin and specific post-translational histone modifications [1]. In *Drosophila*, PcG proteins are recruited to chromatin through Polycomb Response Element (PRE) sequences which can be located several tens of kilobases away from their target genes. PREs recruit the PRC2 complex, which trimethylates lysine 27 of histone H3 (H3K27me3), a mark that is recognized by PcG proteins of the PRC1 complex, such as PC and PH [2], to bring about gene silencing [3]. H3K27me3 and its associated silencing mediated by Polycomb group (PcG) proteins play a crucial role by repressing key developmental regulators in embryonic stem cells [4], [5]. Recent genome wide analyses of the chromosomal distribution of PcG proteins and their associated histone mark H3K27me3 suggest a hierarchical organization [6], [7]. The first level consists of short individual regions bound by PcG proteins. These binding peaks include the previously characterized PcG response elements (PREs), namely DNA regions that are necessary and sufficient to recruit PcG proteins and silence flanking genes [5], [8]. The second level of this organization involves the clustering of individual PREs into large Polycomb domains marked with histone H3K27me3 and, to a lower extent, by the PC protein. Within these genomic regions, PREs may interact to form three dimensional (3D) structural domains inside the cell nucleus, as has been recently demonstrated by chromosome conformation capture for individual PREs of the BX-C locus [9]. Moreover, large Polycomb domains may mediate long-range contacts to form a Polycomb network inside the cell nucleus, since PREs have been shown to contact loci at long-distance on the same chromosome or other chromosomes [10]–[14]. These contacts are functionally regulated because the frequency of interaction depends on PcG proteins [12], [14] as well as on proteins involved in RNAi and in chromatin insulator function [14], [15].

Within the cell nucleus, the PC protein does not have a uniform distribution, but is organized in nuclear foci called PC bodies [16], [17]. PcG-mediated gene silencing occurs within PC bodies [15] and it was proposed that PcG components located in PC bodies may mediate chromatin condensation of their target genes [18], [19]. For instance, *Fab-7*, a PRE-containing region controlling the expression of the gene *Abd-B*, is found within PC bodies in the head of *Drosophila* embryos where *Abd-B* is repressed, whereas in the posterior part, where *Abd-B* is expressed, *Fab-7* is located outside PC bodies [9], [10]. Fluorescence recovery after photo-bleaching experiments show that PcG proteins exchange rapidly in *Drosophila* and mammalian embryonic stem cells [20], [21], demonstrating that, within PC bodies, there is a dynamic exchange between PcG proteins in the nucleoplasm and those located within PC bodies. This suggests that PcG protein binding to their chromatin targets may induce the formation of nuclear bodies. An alternative possibility is that PC bodies might be specialized nuclear structures to which PcG targeted genes convene in order to be silenced. In any case, the specific interaction between PREs implies that they move within the cell nucleus.

Time-lapse imaging of specific chromosome sites tagged with fluorescently labeled topoisomerase II showed that these chromosome sites undergo substantial Brownian motion, but that each site is confined to a sub-region of the nucleus [22]. By using the Lac repressor/lac operator system, the motion of chromosomal loci has been described as consistent with a random walk [23]. In mammalian cell lines, the movement of chromatin loci depends on their nuclear localization since loci closer to the nucleoli and the nuclear periphery are more constrained than other loci [24]. Cell cycle phase and differentiation also influence the motion of chromatin loci. For example, in yeast interphase nuclei early and late origins of replication are highly mobile in G1 and become constrained in S phase. In contrast, telomeres and centromeres display constrained motion in both G1 and S phase [25]. In *Drosophila* spermatocytes, multiple regimes of constrained chromosome motion have been documented during progression through G2 [23]. Another study demonstrated that the movement of chromatin is more constrained in differentiated cells of eye imaginal discs [26]. Interestingly, long-range directional motion of specific loci was identified upon transcriptional induction in mammalian cultured cells. Unidirectional migration of an interphase chromosome site from the nuclear periphery to the interior was observed 1–2 h after targeting a transcription activator to this site, and actin and nuclear myosin I were shown to regulate this migration [27]. Similarly, U2 genes are recruited towards a relatively stably positioned Cajal Body after transcriptional activation, and this long-range chromosomal motion is perturbed by a dominant negative mutant of β-actin [28]. Time-lapse imaging has demonstrated that nuclear bodies also move in the nuclear volume of cultured cells. For example, the motion of Cajal Bodies has been described by anomalous diffusion, alternating between chromatin association and diffusion within the interchromatin space [29]. Moreover, the motion patterns of different types of nuclear bodies were found to be similar, suggesting that their mobility may reflect the dynamics and accessibility of the chromatin environment [30]. However, the motion of PC bodies has never been described.

Since interactions between PREs depend on cell type and chromatin dynamics is affected by cell differentiation, we hypothesized that the formation of PC bodies and their nuclear positioning might depend on developmental processes. We therefore studied the formation and motion of PC bodies during fly embryogenesis. We first analyzed the relation between the size of genomic PC domains and PC enrichment in PC bodies. We then compared the motion of PC bodies with the movements of chromatin domains during *Drosophila* embryogenesis. We found that large PC bodies contain the homeotic complexes, whereas smaller PC domains are located in PC bodies containing less PC. During early embryogenesis, both PC and PH progressively accumulate within PC bodies and the kinetics of PC slows down. The motion of both PC bodies and chromatin domains follows two types of regimes. Firstly, each chromatin domain and PC body has its own fast motion confined in volumes much smaller than that of chromosome territories. Secondly, long-range coordinated motions of several chromatin domains and PC bodies demonstrate the presence of “higher-order” nuclear structures. Finally, both motion regimes progressively slow down during embryogenesis suggesting a correlation between the flexibility of chromatin structures and the potential for cell differentiation.

## Results

### The PC content within PC bodies correlates with the linear size of PC domains

**Figure 1:**
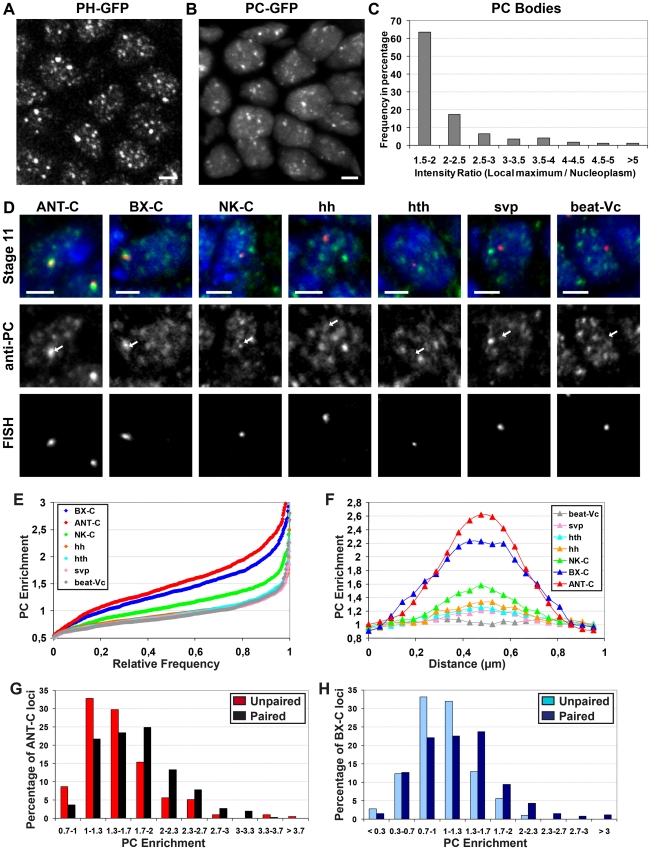
PC enrichments within PC bodies depend on the length of genomic domains coated by PC. A–C: Non-normal distribution of PC enrichment within PC bodies. 3D images of embryos expressing PH-GFP (A) or PC-GFP (B) show intense and faint PC bodies. The scale bar is 2 µm. Histogram showing the distribution of PC enrichment within PC bodies (C). D–H: The amount of PC within a PC body depends on the genomic length of regions bound by PC. 2D images of Immuno-FISH experiments performed with probes located in large (∼400 kb: ANT-C and ∼340 kb: BX-C), medium (∼200 kb: NK-C) or small (∼50 kb: hh, hth and svp) genomic regions coated by PC, as well as probes directed against beat-Vc which is not coated by PC (D). The scale bar is 2 µm. Cumulative histograms of PC enrichment measured within the FISH volumes (N>500 for each FISH probe) (E). 1-µm profiles of PC enrichment along lines crossing their corresponding FISH volumes (N>57 for each FISH probe) (F). Histograms showing that homologous chromosome pairing increases the enrichment of PC within PC bodies containing ANT-C (p<0.001, KS test with N>190) (G) or BX-C loci (p<0.001, KS test with N>175) (H).

Previous studies have shown that PC accumulates in discrete nuclear spots called PC bodies in *Drosophila* [16] and mammals [31], [32]. We analyzed living embryos of transgenic flies expressing a PH-GFP fusion protein [33] or a PC-GFP fusion protein [34] during germ band elongation (stage 11). 3D confocal imaging shows that the distribution of PC-GFP co-localizes with immuno-labellings performed with a specific antibody against PH [15] and H3K27me3 (Figure S1A), showing that PC-GFP is correctly targeted to PcG-bound chromatin *in vivo*. Similarly, an immuno-labelling performed with a specific antibody against PC co-localizes with the distribution of PH-GFP (Figure S1B). 3D vizualization of entire nuclei located in the anterior part of embryos shows that both PH-GFP (Figure 1A) and PC-GFP (Figure 1B) accumulate in a few nuclear dots of heterogenous intensity that represent PC bodies. To quantify the enrichment of PC-GFP within PC bodies, we calculated the ratio between the maximum intensity of PC-GFP measured within PC bodies and the average PC-GFP intensity of the cell nucleus, within optical sections collected at stage 11. The distribution of PC body intensities indicates that they form heterogeneous nuclear structures, with few intense PC bodies and greater numbers of low-intensity bodies (Figure 1C).

Interestingly, recent genome wide analyses have shown that PC binds to genomic domains of different linear size, defined as PC domains [6], [7], [35], [36]. To test whether the intensity of PC bodies correlates with the length of genomic regions associated with PC, we performed Immunostaining coupled to Fluorescent In Situ Hybridization (I-FISH) experiments using specific antibodies against PC and DNA probes that hybridize to genomic gene clusters coated with PC. In *Drosophila melanogaster*, the largest genomic domains to which PC binds are the homeotic complexes (ANT-C which is ∼400 kb and BX-C, which is ∼340 kb) located on chromosome 3R, which also contains another large cluster of PC-bound genes (NK-C, which is ∼200 kb large). We designed probes that hybridize with the loci *Antp* (ANT-C), *Abd-B* (BX-C) and *lbl/lbe* (NK-C), as well as probes hybridizing to the genes *hh*, *hth* and *svp* genes, which are within smaller PC domains (∼50 kb). As a negative control, we used probes recognizing *beat-Vc*, which is a silenced gene during embryogenesis [37] that is not coated by PC [7]. To quantify the amount of PC co-localizing with the FISH probes, we calculated the ratio between the average intensity of PC within each FISH volume and the average intensity of PC inside nuclei. In order to homogenize sampling, we focused on epidermal cells in the anterior part of the embryos, where no transcription has been reported for *Antp*, *Abd-B* [38], *lbl/lbe* [39], *hth* [40] or *svp* [41].

Typical examples of their nuclear localization compared to PC labeling are shown in Figure 1D and cumulative ratio histograms were plotted for each probe (Figure 1E). The high proportion (∼90%) of homeotic gene complexes having a ratio of PC enrichment above 1 (Figure 1E) indicates that, during interphase, these two loci colocalize with intense PC bodies in every cell nucleus at developmental stage 11. Indeed, the few cases in which homeotic complexes do not locate within PC bodies correspond to mitotic cells (data not shown), where no PC bodies were observed. Although homeotic complexes always located within PC bodies in interphase cells, this was not the case for the other probes tested, which co-localized with PC bodies in a fraction of the cells. Moreover, the homeotic complexes localize within intense PC bodies because the volume occupied by their FISH probes contains high enrichment of PC (Figure 1E). In contrast, the NK-C locus mainly locates in intermediate PC bodies. Finally, this approach did not find significant differences between the enrichment of PC within the volumes occupied by the probes detecting the smaller PC target regions (*hh*, *hth* and *svp*) and the negative control (*beat-Vc*) (Figure 1E). Since detectable levels of PC are found in most of the nuclear volume and the amount of PC bound to these loci is small, the specific relative enrichment of PC on smaller PC target regions is difficult to discriminate from background nuclear PC levels. However, a specific enrichment of PC on the FISH probes should produce a peak co-localized with FISH signals. To improve this quantification, we thus calculated the average PC profiles along 1 µm lines crossing the FISH signals in 2D images of I-FISH experiments at developmental stage 11. The center of each profile was defined as the pixel displaying the local maximum intensity in the FISH channel and the PC enrichment was normalized by the PC intensity measured in the left and right ends of each profile. This result confirms that the homeotic complexes locate in intense PC bodies. Moreover, the smaller PC-target regions (*hh*, *hth* and *svp*) show a significant increase in the center of the FISH signal, whereas no increase was observed in the negative control (*beat-Vc*) (Figure 1F). This result demonstrates that small genomics regions also localize within PC bodies, but their intensity is much weaker than those observed with large genomic domains. The intermediate PC profile observed on the NK-C locus (Figure 1F) correlates with intermediate genomic length of its cognate PC domain. Taken together, these results indicate that PC enrichment within PC bodies depends on the linear size of PC domains.

The correlation between the Chromatin Immuno Precipitation (ChIP) on chip and the PC body signals suggests that PC bodies mostly originate by PC binding to their target genes, rather than PC target genes moving to preassembled PC bodies in the nucleus. However, it remains possible that PC-bound loci moving in the nucleus meet each other and associate. In this case, their association should increase the amount of PC within the corresponding PC body. Since homeotic complexes are always found in intense PC bodies at stage 11, homologous chromosome pairing should increase the amount of PC within the PC bodies containing the homeotic complexes. We compared the amount of PC on homeotic complexes within nuclei containing one (Paired) or two (Unpaired) FISH spots (Figure 1G–1H). This comparison clearly shows that homologous chromosome pairing increases the amount of PC within both ANT-C (Figure 1G) and BX-C (Figure 1H) compared to the unpaired situation. Therefore, the amount of PC within one body depends on the length of the genomic regions bound by PC and on the association between them.

### Progressive enrichment of PC and PH within PC bodies during early embryogenesis

**Figure 2:**
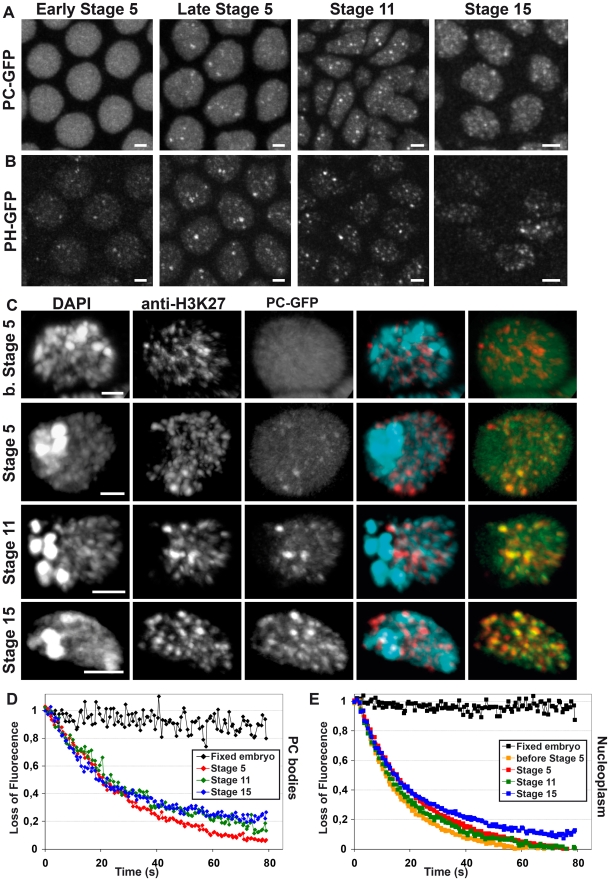
Progressive enrichment of PC-GFP and PH-GFP within PC bodies during embryogenesis. A: 3D visualization of living embryos expressing PC-GFP at different developmental stages. B: 3D visualization of living embryos expressing PH-GFP at different developmental stages. During early development, both PC-GFP and PH-GFP form faint PC bodies and their intensity progressively increases during mid-embryogenesis. C: 3D visualization of fixed nuclei taken from embryos expressing PC-GFP and immuno-labeled with anti-Histone H3K27me3. At any developmental stage, H3K27me3 is distributed in numerous small dots, which never co-localize with DAPI-dense regions. Before stage 5, PC bodies are difficult to observe because of nucleoplasmic PC-GFP but, starting from stage 5, accumulation of PC-GFP is observed in nuclear structures containing H3K27me3. Bars measure 2 µm. D–E: FLIP experiments monitoring the changes of PC-GFP kinetics during embryogenesis. FLIP experiments were performed on embryos expressing PC-GFP by collecting 2D images every 1.3 s for 80 s. A fixed spot of about 500 nm was bleached for 0.3 s every two images during entire time-lapse experiments. The loss of fluorescence inside the most intense PC body of each nucleus (D) and the loss of fluorescence within the nucleoplasm (E) globally slow down during embryogenesis (N>19 for each developmental stage).

In order to analyze the dynamics of PC bodies during embryonic development, we characterized the 3D localization of PC-GFP (Figure 2A) and PH-GFP (Figure 2B) in entire nuclei located in the anterior part of living embryos. Because PC is located in the same genomic domains as H3K27me3 in ChIP on chip experiments [7], [35], the localization of PC-GFP was also compared to the distribution of H3K27me3 at different embryonic stages (Figure 2C). Before and at the beginning of stage 5 (cellularization of the blastoderm), only faint accumulations of PC-GFP and PH-GFP could been seen inside nuclei (Figure 2A–2C). During later develomenptal stages, enrichments of PC and PH within PC bodies progressively increase (Figure 2A–2C). The overlay of PC-GFP and H3K27me3 channels (Figure 2C and Figure S1A) and the line-profile (Figure S1A) clearly show that PC bodies strongly co-localize with chromatin domains containing histone H3K27me3. Noticeably, PC bodies never locate within pericentric heterochromatin which is easily identified by very intense DAPI staining. Moreover, DAPI is not uniformly distributed within euchromatin and histone H3K27me3 is surprisingly found in DAPI-poor regions of euchromatin (Figure 2C). Consistently, DAPI and histone H3K27me3 profiles do not correlate (Figure S1C), suggesting that PC bodies do not represent the most condensed part of *Drosophila* euchromatin.

Since the amount of PC within bodies globally changes during embryogenesis, we performed I-FISH experiments at three embryonic stages (6–7, 11 and 15) to monitor the change of PC enrichment on the genomic probes. To quantify the amount of PC co-localizing with the FISH probes, we calculated a ratio between the average intensity of PC within each FISH volume and the average intensity of PC inside nuclei. Cumulative histograms were plotted for each probe at stages 6–7, 11 and 15 (Figure S2B–S2D) and typical examples of their nuclear localization compared to PC labeling are shown in Figure S2A. Homeotic gene complexes were located in the most intense PC bodies at all developmental stages. Note that the relative PC content at homeotic complexes decreased in embryos at stage 15 but, since the average intensity of PC bodies in the nuclei increases at this stage, this might rather reflect increased intensity of other PC bodies than loss of PC from homeotic PC bodies. Small genomic regions were found in weak PC bodies throughout embryogenesis. Although the NK-C locus always locates in PC bodies of intermediate intensity, the enrichment of PC on the NK-C locus increases during development. Therefore, the intensity of PC bodies associated with specific loci such as NK-C might also be regulated during developmental progression.

If PC bodies are specific chromatin structures to which PC binds, their PC content could depend on kinetics of the protein. By using FRAP, it has been shown that PC rapidly exchanges in living Drosophila [20], demonstrating that PC can easily reach any chromatin target inside the nuclear volume. Since PC bodies were faint during very early embryogenesis (before stage 5), one possibility is that the kinetics of PC-GFP may change during development. To test this hypothesis, Fluorescence Loss In Photobleaching (FLIP) experiments were performed during embryogenesis. At each stage, we monitored the average decrease of the maximum and mean intensities of fluorescence within cell nuclei over time. The maximum intensity allows measurement of the fluorescence decrease within the most intense PC body of each cell nucleus, whereas the decrease of mean intensity mainly relies on PC-GFP kinetics in the nucleoplasm (Figure 2D–2E). From stage 5 to 11, PC-GFP kinetics inside the nucleoplasm are quite similar (Figure 2E), whereas the decrease of the maximum intensity slows down between stages 5 and 11 (Figure 2D). This indicates that the residence time of PC-GFP within PC bodies increases during early embryogenesis. PC-GFP kinetics inside the most intense PC body does not change between developmental stages 11 and 15 (Figure 2D), whereas the kinetics of the nucleoplasmic fraction slows down (Figure 2E). This result is consistent with a decrease of the nucleoplasmic fraction of PC and is related to the increase of PC body intensities during development. Together, these data suggest that PC is progressively recruited to PC bodies and becomes more stably associated with them. Since PC bodies correspond to target genes, this implies that the association of PC with their targets is progressively stabilized during embryogenesis.

### The motion of both PC bodies and chromatin domains does not follow a random walk

**Figure 3:**
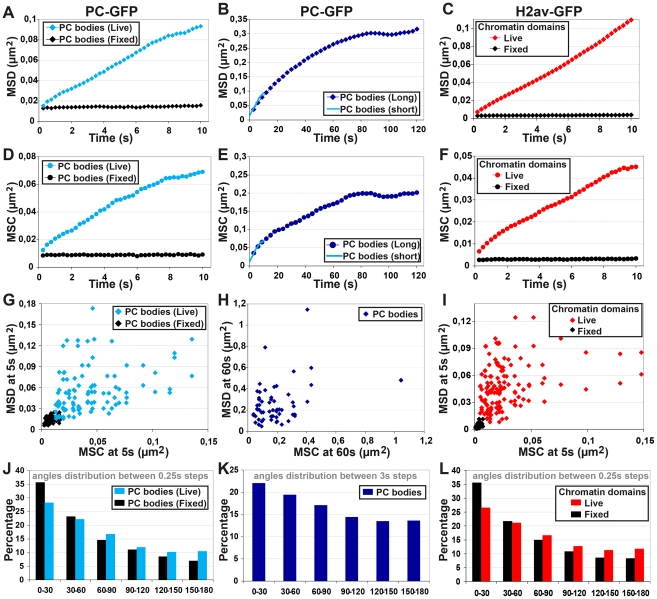
Both PC bodies and chromatin domains display complex motion within the cell nucleus. A–F: Quantification of the motion of PC bodies and chromatin domains. Mean square displacements (MSD) describing the motion of PC bodies (PC-GFP: A–B) and chromatin domains (H2Av-GFP: C). Mean square changes (MSC) illustrating the motion of PC bodies (PC-GFP: D–E) and chromatin domains (H2Av-GFP: F). Time-points were collected every 250 ms for short time-lapse experiments (A, C, D, F) and every 3 s for longer ones (B and E). G–I: Absence of correlation between MSD and MSC. Scatter-plots for the MSD of each PC body or chromatin domain and their corresponding MSC, computed for motions of 5 s (G and I) or 60 s (H). J–L: Narrow angles are over-represented in tracks of PC bodies and chromatin domains. Histograms presenting the occurrence of angles calculated between three consecutive time-points in tracks of PC bodies (J and K) or chromatin domains (L).

Although the PC enrichment within PC bodies during early embryogenesis matches with the genome wide localization of PcG proteins [4], [5], recent studies have demonstrated local and long-range spatial networks of PREs [9], [10]. This suggests a scenario whereby motion of PC bodies in the cell nucleus may promote encounters between different PcG-target loci. Previous studies have shown that chromatin loci undergo constrained random walks confined to a sub-region of the nucleus corresponding approximately to the size of one chromosome territory [22], [23], [25]. In order to characterize the motion of PC bodies, we performed time-lapse experiments to analyze PC-GFP (Figure 3; FIGURE S3 and S4). In particular, at developmental stage 11 we collected 2D images every 250 ms for 15 sec (Figure 3; FIGURE S3B and Video S1). The Mean Square Displacement (MSD) increases almost linearly with time, suggesting that PC bodies follow a random walk within the live cell nucleus, whereas in control experiments of fixed nuclei the bodies do not move because their MSD curve is flat (Figure 3A). To measure the volume in which PC bodies are confined, we also performed longer time-lapse experiments by acquiring 3 µm-thick volumes every 3 s for 3 min. We analyzed their tracks without accounting for motion in the Z-axis (Figure 3 and Video S2). The first time-points of the corresponding MSD curve perfectly match the curve calculated from short time-lapse experiments (Figure 3B). Moreover, a plateau corresponding to a confined volume with radius 560 nm is clearly observed between 1 and 2 min. This confinement volume of PC bodies approximates to that of a chromosome territory (710 nm; approximated by dividing the nuclear surface at this developmental stage by the number of chromosome arms without taking into account the chromosome 4 which represents less than 1% of the fly genome).

However, visualization of movies monitoring the motion of PC bodies suggests that these nuclear structures are constrained within smaller volumes (Videos S1 and S2) and we decided to not only use the MSD criterion to characterize their motions. Indeed, in a random walk regime, any given angle has the same probability to occur between three consecutive time-points of a given tracked object. To specifically test this, we systematically calculated angles between two displacements of the same duration. The histograms of their occurrence clearly indicates that the motion of PC bodies is mainly composed of narrow angles (0° to 60°) (Figure 3J–3K), even for durations as short as 0.5 sec. This result is inconsistent with random walk motion of PC bodies and suggests that a constraint operates at a much smaller scale than the one described with the MSD analysis. Finally, the Mean Square Changes monitoring the variation of inter-distances between two PC bodies (MSC) were also calculated for both short and long tracks (Figure 3D and 3E). Although their results were similar to MSD measurements, their correlations are weak (Figure 3G and 3H), indicating that MSD or MSC curves alone are not sufficient to fully describe the motion of PC bodies. Taken together, the weak correlation between the MSD and MSC data and the over-representation of narrow angles demonstrate that PC bodies do not simply follow a random walk in a constrained nuclear volume.

We then extended the analysis to the motion of chromatin domains by time-lapse microscopy of the H2Av-GFP histone variant in embryos. H2Av behaves genetically as a PcG gene and mutations in H2Av suppress position effect variegation [42]. Genome wide analysis indicates that 85% of Drosophila coding genes contain at least one H2Av nucleosome [43] and H2Av is associated with both transcribed and nontranscribed genes in polytene chromosome bands and interbands [44]. H2av-GFP histone forms numerous small domains within euchromatin and its 3D localization correlates with DAPI staining in euchromatin, whereas it is depleted from part of pericentric heterochromatin (Figure S1D). H2Av-GFP euchromatin domains are visible during entire time-lapse experiments and therefore can be used to monitor the bulk motion of condensed *Drosophila* euchromatin (Figure S5B and Video S3). 2D images of embryos expressing H2Av-GFP were collected every 250 ms for 15 sec at developmental stage 11 and calculation of MSD and MSC gave similar results to the ones observed with PC bodies. Inaccurate 3D segmentation of chromatin domains prevented us from analyzing longer time-lapse experiments. Similarly to PC-GFP, MSD (Figure 3C) and MSC (Figure 3F) curves linearly increased with time but the MSD of an individual track correlates only weakly with its corresponding MSC (Figure 3I) and the motion of chromatin domains is mainly composed of narrow angles (0° to 60°) (Figure 3L). Taken together, these results suggest that PC bodies and chromatin domains behave similarly and that their motion cannot be simply described by random walk within a sub-nuclear volume.

### Evidence for coordinated motion of condensed chromatin domains and PC bodies

**Figure 4:**
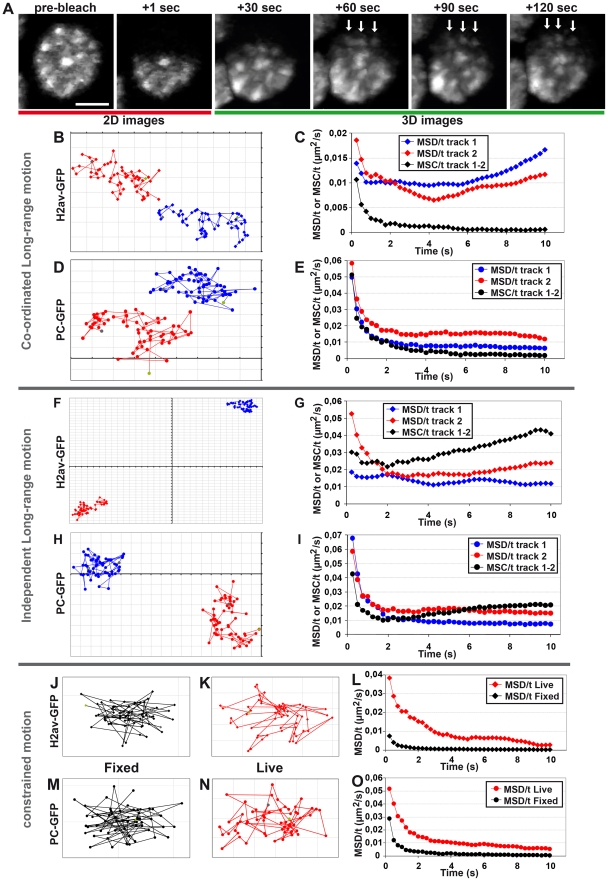
Evidence for coordinated motion of chromatin domains and PC bodies. A: A cell nucleus expressing H2Av-GFP was half-bleached and subsequent time-lapse movies show obvious coordinated motions of several chromatin domains (arrows). The scale bar is 2 µm. B–E: Coordinated long-range motion of chromatin domains and PC bodies. Tracks of two chromatin domains (B) or PC bodies (D) inside one nucleus and their corresponding MSD/t and MSC/t curves over time (C and E). In both cases, the two tracked structures coordinately move because their MSD/t curves are much higher than their corresponding MSC/t curves (C and E). F–I: Independent long-range motion of chromatin domains and PC bodies: Tracks of two chromatin domains (F) or two PC bodies (H) inside one nucleus and their corresponding MSD/t and MSC/t curves over time (G and I). The corresponding MSC/t curves indicate a faster motion than MSD/t curves, indicating that the two tracked structures independently move. J–O: Constrained motion of chromatin domains and PC bodies. Single tracks of chromatin domains (J and K) or PC bodies (M and N) with their corresponding MSD/t curves over time (L and O). Although a similar decrease is observed in fixed and living cells (L and O), both PC bodies and chromatin domains move since their corresponding MSD/t curves are higher in living embryos than after fixation. The MSD/t curves observed in fixed cells depend on the accuracy of image segmentation required to calculate the tracks of chromatin domains and PC bodies (J and M). In living embryos, some tracks of chromatin domains (K) and PC bodies (N) seem to loop around a preferential position, which explains why their corresponding MSD/t curves rapidly reach an asymptote approaching zero (L and O). Each grey line is spaced by 100 nm (B, D, F, H, J, K, M and N).

To better characterize chromatin motion, we bleached half of selected nuclei in the anterior part of embryos expressing histone H2Av-GFP at developmental stage 11 and 3D images were recorded every 3 sec for 3 min. During the duration of the experiments, only a small fraction of H2av-GFP recovers within the bleached area (data not shown). Therefore if chromatin moves by diffusion, the border between bleached and unbleached areas should progressively blur and disappear because chromatin from the unbleached area should progressively move towards the bleached area. In contrast, we found that distinct chromatin domains move from the unbleached region to the bleached volume and most of the time, several chromatin domains simultaneously undergo similar motion (Figure 4A and Video S4). For example, several distinct chromatin domains (arrows in Figure 4A) move coordinately over a distance greater than 500 nm. These data show that chromatin is organized in small 3D domains that frequently undergo coordinated long-range motion.

In order to distinguish the motion of individual PC bodies or chromatin domains from the coordinated motion of multiple bodies and chromatin domains, we analyzed pairs of tracks. A careful examination of multiple tracks often allowed us to find pairs of tracks displaying a simultaneous long-range motion (Figure 4B and 4D). We thus plotted the MSD/t over time, in order to characterize the motion of individual tracks. The comparison between two tracks was made by plotting the MSC/t over time (Figure 4C and 4E). Consistent with the coordinated long-range motion of chromatin domains, the plot of MSC/t over time rapidly converges towards 0, whereas MSD/t curves globally stop their decrease after 3 s. This type of behavior indicates that most of the displacement measured with MSD/t curves does not relate to the motion of each individual tracked object, but mainly relies on the displacement of a higher-order nuclear structure containing the two tracked objects. The simultaneous coordinated motion of the two tracked objects also explains the absence of correlation between MSD and MSC values because this motion regime is characterized by weak variation of inter-distance (low MSC values) between two tracked objects that may possess high MSD values due to long-range motion of a higher-order nuclear structure containing them. Another instance of absence of correlation between MSD and MSC curves could also be observed in cases when the MSC is larger than the MSD. This indicates that the two tracked objects move independently (Figure 4F–4I). This typically occurs when each track belongs to distinct higher-order nuclear structures undergoing different long-range motions.

### Chromatin domains and PC bodies undergo rapid, locally constrained motion

About half of the tracked objects stay within constrained volumes close to their initial positions during the 15 sec of the time-lapse (Figure 4J–4O). Interestingly, the MSD/t of constrained tracks decreased rapidly over time, asymptotically tending towards 0 (Figure 4L and 4O), suggesting that the motion of these PC bodies and chromatin domains rapidly reaches the limit of their confinement volumes. Comparison between tracks collected in fixed embryos (Figure 4J and 4M) and constrained tracks of live objects (Figure 4K and 4N) clearly demonstrates that live objects displaying constrained tracks are moving, since their MSD/t curves are higher than the ones corresponding to fixed objects. Furthermore, the analysis of constrained tracks collected in live cells (Figure 4K and 4N) shows that the tracked objects do move away from their initial positions, but then consistently come back, close to the point of origin. Therefore, the MSD/t values calculated after short times depend on the range by which these objects move away from their original position in these constrained tracks. Interestingly, the MSC/t curve between two objects having a coordinated motion (Figure 4C and 4E) is similar to the MSD/t curves corresponding to constrained tracks (Figure 4L and 4O). This indicates that the inter-distance variation between two objects undergoing a coordinated long-range motion is similar to that of the constrained motion regime. Therefore, all PC bodies and chromatin domains undergo local, highly constrained motion. Taken together, these results demonstrate that motion of PC bodies and chromatin domains is composed of two components: the first is constitutive and consists of locally highly constrained displacements, and the second regime corresponds to occasional long-range movements affecting higher-order nuclear structures.

**Figure 5:**
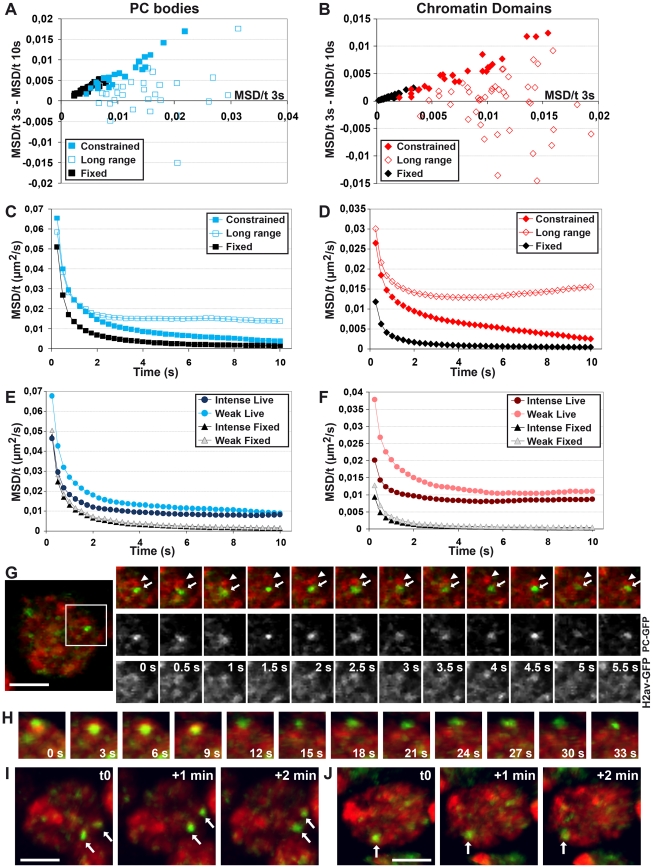
Characterization of the motion of PC bodies and chromatin domains. A–B: Discrimination between tracks of PC bodies (A) or chromatin domains (B), showing only constrained motion and the ones displaying both constrained and long-range motions. A correlation between the MSD/t after 3 s and the difference of MSD/t between 3 s and 10 s (full squares and diamonds) indicates only one component in the motion, whereas long-range motion disturbs this correlation in other cases (empty diamonds and squares). C–D: Quantification of constrained and long-range motion. MSD/t curves over time, monitoring the constrained motion of PC bodies (C) or chromatin domains (D), rapidly decrease and reach asymptotes towards zero, indicating that both structures rapidly reach the limits of their volume of confinement when no long-range motion is observed. In contrast, MSD/t curves over time of PC bodies (C) or chromatin domains (D) displaying both constrained and long-range motions rapidly decrease and then stay approximately horizontal. E–F: The intensity of PC bodies and chromatin domains influences their motion. MSD/t curves over time of intense PC bodies (E) or intense chromatin domains (F) stay below the ones of weak PC bodies or chromatin domains (p<0.001, t-test on log values reached after 1 s). G–J: Chromatin domains and PC bodies form distinct structures undergoing occasional coordinated long-range motion. 2D images from an embryo at developmental stage 15 expressing both H2B-RFP and PC-GFP (G) present an example of independent motion of PC bodies (arrow) compared to surrounding chromatin (arrowhead). 4D images illustrate both distinct local motion of PC bodies from the surrounding chromatin visualized with H2B-RFP (H) and simultaneous coordinated motion of both (arrows in I and J).

To discriminate between locally constrained motion and occasional long-range motion, we computed scatter-plots between the difference of MSD/t between 3 and 10 s and the MSD/t at 3 s for both PC bodies and chromatin domains (Figure 5A and 5B). In the tracks where these two values correlate, the movement only depends on the locally constrained regime. On the other hand, a lack of correlation indicates that both locally constrained and long-range regimes influence the motion. In order to only analyze the locally constrained motion, we plotted the average of all the MSD/t curves in which these two values correlate (Figure 5C and 5D). Consistent with the typical examples shown in Figure 4, the MSD/t over time curves rapidly tend asymptotically towards 0, indicating that both chromatin domains (Figure 5D) and PC bodies (Figure 5C) reach the limits of the volume in which they are confined, after approximately 5 s. In the absence of long-range motion at developmental stage 11, chromatin domains move on average within volumes of radius 160 nm, whereas the motion of PC bodies is confined within volumes of radius 200 nm. By averaging the MSD/t curves in which the difference of MSD/t between 3 and 10 s does not correlate with the MSD/t at 3 s, we analyzed the movement of chromatin domains and PC bodies in tracks displaying both local constrained and long range motions (Figure 5C and 5D). The MSD/t curves of both chromatin domains and PC bodies reach horizontal lines clearly above 0, after a short rapid decrease (Figure 5C and 5D). Although a random walk would produce such profiles, the sum of local constrained motion and directional long-range motion could also lead to horizontal MSD/t curves.

### The motion of chromatin domains is inversely related to the amount of H2Av and PC

In order to test whether PC bodies containing more PC-GFP move less than weaker PC bodies, we analyzed the relation between the enrichment of PC-GFP and their respective MSD/t. We tracked two PC bodies per nucleus, defining two groups: one containing the most intense PC body tracked in each nucleus, while the weakest PC body was placed in the second group. The most intense PC bodies clearly move less than the weakest ones (Figure 5E). To test whether more intense chromatin domains move less than weaker ones, we used the same approach. We compared the motion of two groups of chromatin domains: one containing the most intense H2Av-GFP domains tracked in each nucleus, and the second group contains the weakest ones tracked in the same nuclei. Again, chromatin domains containing more H2Av-GFP move less than the weakest ones (Figure 5F). Importantly, the dependence of local constrained motion on an inner parameter like the content in H2Av-GFP or PC-GFP indicates that each chromatin domain or PC body has its own individual constrained motion. This is in contrast to long-range displacement, which influences several chromatin domains at a time, leading to their coordinated motion.

### Chromatin domains and PC bodies belong to larger nuclear structures

To compare the motion of PC bodies with that of bulk chromatin domains, time-lapse experiments were performed on fly embryos expressing both PC-GFP and Histone H2B-RFP. We analyzed their motions at developmental stage 15 by collecting 2D images every 500 ms for 15 sec (Figure 5G and Video S5) and by acquiring 3 µm-thick volumes every 6 s for 3 min (Figure 5H–5J and Video S5). Consistent with the observation in fixed cells (Figure 2C and Figure S1C), PC bodies often locate adjacent to chromatin domains (Figure 5G–5J). Furthermore, the relative position of PC bodies compared to adjacent chromatin domains can rapidly change over time (Figure 5G), and chromatin around a given PC body can modify its organization (Figure 5H). Therefore, PC bodies can move independently of their surrounding chromatin. Nevertheless, the position of PC bodies inside the nucleus is globally conserved during the tracking experiments (Figure 5I–5J and Video S5). Indeed, 4D tracking of both PC bodies and chromatin domains for 3 min demonstrates that long-range motion can involve both kinds of structures simultaneously (Figure 5I–5J), suggesting that they correspond to movements of “higher-order” structures containing several chromatin domains and PC bodies.

To analyze the motion of PC bodies during longer time-scales, living embryos expressing PC-GFP (Video S6) or PH-GFP (Video S7) were observed by collecting 1 volume every 10 s for 30 min. At this time-scale, the pattern of PC bodies within the cell nucleus is globally conserved. Associations and dissociations of PC bodies were observed and they seem to rely on long range motion occurring in the range of 10 to 30 sec (Figure S4). Indeed, time-lapse imaging demonstrates that some PC bodies are composed of several sub-structures which can split and merge (Figure S4A). Interestingly, stable association between two PC bodies can also occur during mid-embryogenesis (Figure S4C). However, association and dissociation events appear to be more dynamic during early embryonic development (Figure S4B, S4D and S4E) and were not observed during later development.

### Both constrained and long-range motions of chromatin domains and PC bodies decrease during embryogenesis

**Figure 6:**
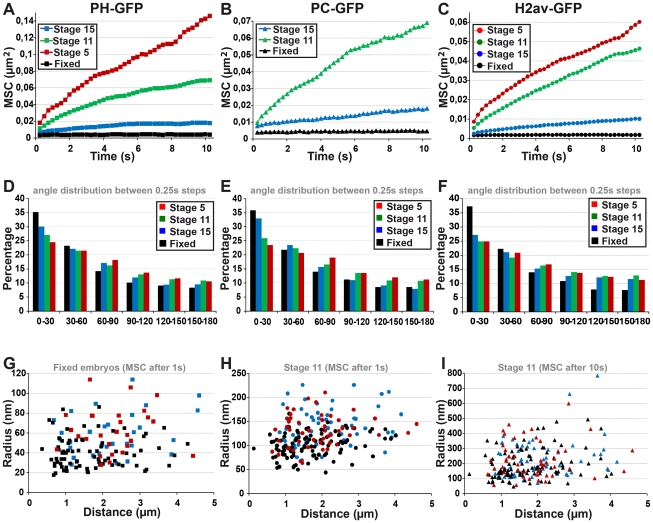
The fast local motion of PC bodies is less constrained than that of chromatin domains. A–C: Mean square change (MSC) monitoring the motion of PC bodies (PH-GFP: A and PC-GFP: B) and chromatin domains (H2Av-GFP: C) during embryogenesis. The kinetics of PC bodies monitored by PC-GFP or PH-GFP are similar throughout embryogenesis. Although PC bodies and chromatin domains similarly slow down during development, chromatin domains consistently move less than PC bodies (p<0.001, KS test calculated with MSC values reached after 1 s). D–F: Histograms presenting the frequency of angles calculated between three consecutive time-points in tracks of PC bodies (PH-GFP: D and PC-GFP: E) or chromatin domains (F). Narrow angles are over-represented in tracks of PC bodies and condensed chromatin domains throughout embryogenesis. G–I: Scatter plots comparing the average radius of the volume in which PC bodies (PH-GFP: blue points and PC-GFP: red points) or chromatin domains (black points) move, with the distance between the two objects tracked.

The amount of PC and PH within PC bodies changes during embryonic development, as well as the nuclear distribution of H2Av-GFP, which forms fewer, larger and more intense chromatin domains in late embryonic stages. To analyze whether these changes are related to the motion of chromatin domains (Figure S5A–S5C) and PC bodies (Figure S3), time-lapse experiments were performed at developmental stages 5, 11 and 15. Since PC bodies contain both PC and PH, both PC-GFP and PH-GFP can be used to monitor their motion. Although PH-GFP can be used to track PC bodies, segmentation of the cell nucleus was impossible due to the weak fraction of PH-GFP inside the nucleoplasm (Video S8). Therefore to compare time-lapse experiments done with PC-GFP or PH-GFP, we focused on MSC curves which only rely on the variation of distance between two objects and do not require the tracking of cell nucleus movements. At developmental stage 5, two PC bodies could not be simultaneously followed in time-lapse experiments using PC-GFP because of the high nucleoplasmic background of this protein. We therefore used only PH-GFP at this stage. As expected, the motion of PC bodies measured by using PH-GFP and PC-GFP at stage 11 or 15 is highly similar (Figure 6A and 6B). We observe a clear, progressive decrease in PC body mobility on developmental progression. A similar decrease is also observed for condensed euchromatin domains (Figure 6C). Both nuclear structures undergo locally highly constrained displacements at all measured developmental stages, because narrow angles (0° to 60°) between 0.25 sec steps are always over-represented (Figure 6D–6F). Interestingly, condensed euchromatin domains move less than PC bodies (Figure 6A–6C). To illustrate this point, we computed the average radius of the volume in which PC bodies or chromatin domains move during 1 sec. The results obtained in fixed embryos measure the accuracy of these tracking experiments, which is slightly higher in chromatin domains than in PC bodies (Figure 6G). However, the difference observed in living embryos at developmental stage 11 significantly increases, indicating that PC bodies move within a larger nuclear volume compared to chromatin domains (Figure 6H). When the same computation is applied to a time of 10 seconds, the volume in which PC bodies move does not significantly differ from that of chromatin domains (Figure 6I), consistent with the influence of long-range motion on both nuclear structures on longer time-scales.

In nuclei where half of the volume is bleached for H2Av-GFP fluorescence, distinct chromatin domains clearly move from the unbleached area to the bleached volume at all embryonic stages. Moreover, coordinated long-range motions are clearly identified during late embryogenesis (Figure S5D–S5F and Video S4). Since the motion of both chromatin domains and PC bodies does not match with simple diffusion within a sub-nuclear volume at any embryonic stage, MSD calculation was required to discriminate between locally constrained and long-range coordinated motion. This analysis showed that the velocity of both PC bodies (Figure S6A–S6B) and chromatin domains (Figure S7A–S7B) decreases during embryogenesis, and both nuclear objects move in smaller volumes during late embryogenesis compared to the ones measured at early developmental stages. As expected, MSD only weakly correlates with MSC for both PC bodies (Figure S6C–S6E) and chromatin domains at any developmental stage tested (Figure S7C–S7D).

**Figure 7:**
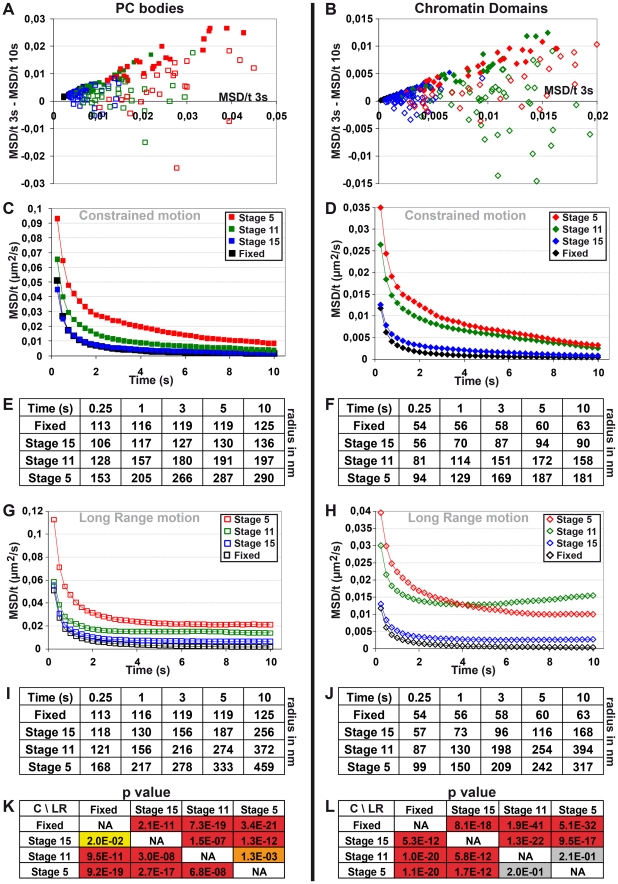
Both constrained and long-range motions of PC bodies and chromatin domains decrease during embryonic development. A–B: Scatter-plots of the MSD/t after 3 s and the difference of MSD/t between 3 s and 10 s for PC bodies (A) and chromatin domains (B). Full diamonds and squares correspond to tracks showing only constrained motion, whereas empty diamonds and squares correspond to tracks displaying both constrained and long-range motions. C–F: Constrained motion of PC bodies and chromatin domains decreases during embryonic development. MSD/t curves over time monitoring the constrained motion of PC bodies (C) or chromatin domains (D) at developmental stages 5, 11 and 15. Tables presenting the corresponding radius of confinement of PC bodies (E) and chromatin domains (F). G–J: Long-range motion of PC bodies and chromatin domains also decreases during embryonic development. MSD/t curves over time monitoring long-range and constrained motions of PC bodies (G) or chromatin domains (H) at developmental stages 5, 11 and 15. Tables presenting the radius of the volumes in which PC bodies (I) and chromatin domains (J) move. K–L: Tables of p-values calculated using t-tests on the log values of MSD/t reached after 5 s for PC bodies (K) and chromatin domains (L) (C = constrained tracks; LR = tracks with long-range motion) (red p<0.001; orange p<0.01; yellow p<0.05 and grey p>0.05).

We then separated the tracks showing only constrained motion from the ones displaying both constrained and long-range motions by using scatter-plots between the difference of MSD/t between 3 and 10 s and the MSD/t at 3 s, for both PC bodies and chromatin domains (Figure 7A–7B). The MSD/t curves over time of tracks showing only constrained motion rapidly approach an asymptote towards 0 for both PC bodies and chromatin domains at all stages tested (Figure 7C–7D). Of note, the corresponding volumes of confinement progressively decrease during embryogenesis (Figure 7E–7F). In the absence of long-range motion, PC bodies are confined within surfaces of 290, 200 and 140 nm in radius, and chromatin domains move on average within surfaces of 180, 160 and 90 nm in radius at developmental stages 5, 11 and 15 respectively. Therefore the volumes of confinement of condensed euchromatin domains and PC bodies decrease during development. The average surface of the cell nucleus is 23, 16 and 14 µm^2^ at developmental stages 5, 11 and 15 respectively, which approximately corresponds to chromosome arm territories having a radius of 860, 710 and 670 nm. Consequently, without occasional long-range motion affecting higher-order nuclear structures, both chromatin domains and PC bodies move within volumes much smaller than chromosome arm territories throughout entire embryogenesis. Interestingly, their mobility apparently decreases more than the decrease of the nuclear volume occurring during embryonic development. These results indicate a strongly constrained motion of general and PC-bound chromatin and suggest that the constraints affecting these nuclear structures progressively increase during development.

The comparison of MSD/t at different developmental stages clearly indicates that long-range motion of both PC bodies (Figure 7G and 7K) and chromatin domains (Figure 7H and 7L), also decreases during embryogenesis. “Higher-order” nuclear structures containing both H2Av-GFP chromatin domains and PC bodies move long distances within the nucleus, and are often related to nuclear deformation (Figure S5; Videos S2 and S4). At developmental stage 5, PC bodies moves in volumes larger than 820 nm in radius (Figure S6A–S6B). MSD curves calculated from long time-lapse experiments tracking the motion of PC bodies reaches a plateau after 1–2 min at stages 11 and 15, which corresponds to volumes with a radius of about 560 and 330 nm, respectively (Figure S6A–S6B). Interestingly, intense PC bodies move less than weak ones at the three developmental stages tested (Figure S8A–S8C). Similarly, a significant negative correlation is also observed between H2Av-GFP enrichment within chromatin domains and their mobility at developmental stages 5 and 15 (Figure S8E and S8G). These results suggest that the influence of PC-GFP or H2Av-GFP enrichment on the motion of PC bodies or chromatin domains is not specific to one developmental stage.

### Temperature influences the motion of chromatin domains and PC bodies as well as PC–GFP exchange

To test whether the motion of chromatin domains and PC domains depends on thermodynamic chromatin features, we compared time-lapse experiments on embryos grown at 18°C or 25°C. At 18°C, embryogenesis takes twice as long as at 25°C. The global motion of chromatin domains slows down at 18°C at all developmental stages (Figure S9A–S9C and S9J). Interestingly, independent of developmental stage, the fast local constrained motion also decreases at 18°C, thus suggesting that the volumes of confinement are smaller at 18°C compared to 25°C (Figure S10A–S10C and S10M). Temperature also affects the long-range motion of chromatin domains at developmental stage 11 (Figure S10D–S10F and S10M). In the case of PC bodies, temperature significantly affects movement during early embryogenesis, whereas no effect is detected during late embryogenesis (Figure S9D–S9F and S9J). In contrast to chromatin domains, the own local constrained motion of PC bodies only significantly decreases at stage 5 (Figure S10G–S10I and S10N). Taken together, these results suggest that motion of PC bodies is less sensitive to temperature during embryogenesis, whereas the motion of chromatin domains depends on temperature during entire embryogenesis.

In order to study the effect of temperature on the kinetics of PC-GFP within PC bodies, we performed FLIP experiments in embryos growing at 18°C and their results were compared to the ones obtained at 25°C. The loss of fluorescence slows down within both PC bodies and nucleoplasm at 18°C compared to the ones observed at 25°C at any developmental stage (Figure S9G–S9I and S9K). Therefore, PC-GFP kinetics clearly depends on temperature, demonstrating that PC binding relies on chromatin thermodynamic properties.

## Discussion

In this study, we show that PC bodies co-localize with H3K27me3 and form small nuclear domains of heterogeneous intensity. Surprisingly, PC bodies are found in DAPI poor regions, often adjacent to DAPI and histone-dense euchromatic regions. This result thus indicates that PC bodies are not among the most condensed chromatin portions of the euchromatic part of the genome. This localization of PC bodies is consistent with a previous study with electron microscopy, which has shown that PC is concentrated in the perichromatin compartment of the mammalian nucleus [45]. On the other hand, our data are in apparent contrast with a series of papers reporting PcG protein-dependent chromatin condensation. PcG complexes have been shown to compact chromatin in vitro [46], [47] and reduce DNA accessibility *in vivo* [48]. Moreover, recent works show that PcG proteins are required to maintain compaction of Hox loci in mammalian embryonic stem cells [18] and of the mouse Kcnq1 imprinted cluster [19]. In those studies, condensation has been addressed by measuring either the compaction of nucleosomal fibers in electron microscopy, or the distance between close genomic loci by FISH. It is difficult to relate *in vitro* data to our *in vivo* analysis. In particular, FISH analyses do not directly distinguish between a truly dense 3D organization and other types of conformations, such as a multi-looped architecture that would not necessarily induce an increase in chromatin density. Therefore, PcG target chromatin is probably organized in higher-order 3D structures that involve nucleosome-nucleosome and protein-protein interactions, but the net density of DNA (as seen by DAPI) or histones (as seen by tagged-histone microscopy) is not particularly high in these structures.

### The nature of Polycomb bodies

Earlier studies indicated that PcG proteins rapidly exchange between the nucleoplasm and PC bodies, suggesting that PC bodies consist of a local transient accumulation of PcG proteins in the cell nucleus [20], [21]. Earlier studies have detected the same number of PC bodies inside the nucleus as the number of bands observed on polytene chromosomes, suggesting that PC bodies are formed by PcG proteins binding to their target chromatin [16]. The observed colocalization of PcG target genes with PC bodies in diploid cells confirms this view [9], [15]. An alternative scenario posits that PC bodies could form nucleation sites onto which PcG-target genes move to become silenced. Two lines of evidence from our work suggest the first scenario to be closer to reality. Firstly, we found that the amount of PC within a PC body depends on the linear size of the genomic region coated by PC and H3K27me3. Secondly, the higher enrichment of PC in PC bodies after homologous chromosome pairing strongly suggests that PC bodies are the nuclear counterparts of linear genomic domains identified in genome-wide studies [7], [35] rather than nuclear structures to which Polycomb target genes have to be localized for their silencing.

In the head of embryos, where the *Antp* and *Abd-B* genes are silenced, they localize in large PC bodies in all cell nuclei. In contrast, loci where PC coating is restricted to smaller genomic regions do not always localize within PC bodies in interphase cell nuclei. Interestingly, time-lapse imaging shows that large PC bodies are stable structures that can be visualized in all frames of time series, whereas small PC bodies are apparently less stable because they are not visible in all of the frames. One possible explanation for the lack of colocalization between PC target genes and PC bodies is that small genomic regions may not be coated by PC in every cell. Alternatively, the amount of PC within the PC body in which small genomic regions localize might be too small to be directly observed, and only become visible when several small PC bodies interact together. For instance a previous study showed that a transgene containing only two copies of a PRE could be detected in about 50% of cell nuclei [15].

### Two regimes of chromatin motion: Implications for the establishment of chromatin contacts

Intense PC bodies can be visualized during entire time-lapse experiments, allowing the study of their motion. The interpretation of these time-lapse experiments is not straightforward because the MSD of PC bodies only weakly correlates with the MSC. Interestingly, tracks of PC bodies are mainly composed of narrow angles. The analysis of the motion of chromatin domains containing H2Av-GFP gave similar results, but gave unambiguous evidence for the coordinated motion of several chromatin domains. By using the Lac repressor/lac operator system, two components of chromatin motion in early G2 *Drosophila* spermatocyte nuclei have been reported: a short range motion which occurs in approximately 0.5 µm radius domains, and long-range motion confined to a large, chromosome-sized domain [23]. Another study has also identified a two-regime motion of a chromatin locus inside mammalian nucleus by using a two-photon microscope, which provides high spatial and temporal resolution. This work indicated that chromatin loci undergo apparent constrained diffusion during long periods, interrupted by jumps of 150 nm lasting less than 2 s [49]. However, none of these previous works reported any coordinated motion of adjacent chromatin domains, and therefore they both described the motion of chromatin as being consistent with a random walk.

In our tracking experiments, we realized that the fast regime of motion is tightly constrained within volumes much smaller than chromosome territories. This suggests that any given locus will normally explore a restricted three-dimensional environment in the cell nucleus. Since this applies generally to chromatin at all developmental stages, one can deduce that each genomic locus is likely to locate in the vicinity of neighboring loci in the three-dimensional nuclear space. The prediction is thus that each locus should most frequently contact other loci that are in its linear neighborhood along the chromosome. This behavior matches the results observed in chromosome conformation capture on chip (4C) experiments [10], [50], where each 4C bait had most contacts within few hundred kb to a few Mb of surrounding chromatin. Thus, our results provide a possible scenario for the explanation of these results obtained from large cell populations. Recent studies showed that homeotic gene clusters form an extensive network of contacts with other PcG target loci [10], [11]. This is consistent with our observation of multiple PC body collisions that can be stable for prolonged times in the nucleus. On the other hand, the fact that PC intensity correlates with the linear extension of genomic PC and H3K27me3 domains suggests that PC-mediated associations are relatively rare, at least during embryogenesis.

The slower regime of long-range motion depends on coordinated large-scale chromatin movements that were not documented before. This may depend on the tools used in previous studies. Time-lapse experiments performed by using the Lac repressor/lac operator system only follow one or a few points inside the cell nucleus [23]–[25], [51], limiting the probability to observe coordinated motions, especially in species containing many chromosomes. In contrast, we followed many chromatin domains inside *Drosophila* nuclei and long-range coordinated motions were easily identified when at least two distinct nuclear structures moved simultaneously with a similar trajectory. This motion is directional and chromatin domains and PC bodies can cover up to 1 µm in 10 sec. Different objects having coordinated motion probably belong to the same structure, which suggests that the ensemble of chromatin domains and PC bodies displaying a similar coordinated motion forms a single higher-order nuclear structure. This kind of motion is perfectly consistent with the observation of a chromosome territory, which implies that chromosomes form distinct nuclear structures in interphase cells [52]. A displacement of an entire chromosome, or of a chromosome arm, or a large part thereof, would induce the coordinated motion of all chromatin domains and PC bodies associated to the corresponding chromosome portion.

The few association and dissociation events of PC bodies observed during this work are related to long-range coordinated motion events that affect both chromatin domains and PC bodies. Therefore, gene kissing depending on PcG proteins [12], [13] could rely on large scale chromatin movements which lead to transient fusion of PC bodies, and may be in turn specifically stabilized by interactions among PcG proteins. Moreover, the association and dissociation of PC bodies seems to be developmentally regulated, because dynamic associations and dissociations were observed during early embryogenesis, but are strongly reduced later in development.

### Determinants of Polycomb body and chromatin dynamics

Condensed chromatin domains and PC bodies move in confined volumes much smaller than chromosome territories. This highly constrained motion prevents chromatin domains from dispersing inside the cell nucleus and can explain why chromosomes form chromosome territories in interphase cells. This movement within highly confined volumes implies that some forces prevent chromatin from diffusing within entire chromosome territories. Interestingly, it was shown before that chromatin loci localized in peri-nucleolar areas or within heterochromatin move less than the ones included in euchromatin, and the authors concluded that association of chromatin loci with different nuclear compartments induces specific constraints on their motion [24]. Another time-lapse experiment performed on one *Drosophila* locus flanking a large block of heterochromatin showed that random association of this locus with pericentric heterochromatin is quite stable and decreases its motion [26], [53]. The motion of larger chromatin structures such as heterochromatin or euchromatin domains cannot be addressed by tracking single loci. By analyzing structures larger than individual chromatin loci, the motion of both bulk chromatin domains and of PC bodies seems to be influenced by their respective local enrichment of histone and PC proteins. Therefore, one key determinant of the motion constraint is an inner property of these structures, which is coherent with the concept of self-organization [54].

The most dramatic change of PC body motion occurs during embryogenesis when nuclear volumes strongly decrease, concomitant with a decrease in bulk chromatin motion. Comparison of chromatin motion between early and late G2 *Drosophila* spermatocytes [23] or between undifferentiated and differentiated cells of eye imaginal discs [26] indicated that the volume in which chromatin loci move decreases during differentiation. However, because of the particularly rapid motion of chromatin domains and PC bodies during early embryogenesis, the slowdown of chromatin motion occurring during embryogenesis is higher than the ones previously described during differentiation. Interestingly, the reduction of the volume of constraint during developmental progression suggests a correlation between the flexibility of chromatin structures and the potential for cell differentiation.

It is interesting to note that the motion of PC bodies appears less sensitive to temperature than chromatin domains in late embryos, suggesting that Polycomb proteins may specifically buffer environmental effects such as temperature change. This buffering may be an important determinant of the stability of Polycomb-dependent gene silencing during development. During this work, no other fundamental difference was observed between the motion of condensed chromatin domains and of PC bodies. This apparent absence in specificity is coherent with data implying that PC bodies form molecularly specialized chromatin regions, but suggests that the molecular identity of these structures is not the main determinant of their motion. Interestingly, a previous study has shown that the artificial Mx1-YFP nuclear body exhibits a very similar mobility compared with Promyelocytic leukemia and Cajal bodies [30]. Although being molecularly different, no specific motion of these nuclear bodies was observed, indicating that the motion of nuclear bodies mainly depends on structural issues such as their size and the nuclear volume. During fly embryogenesis, PC bodies and condensed chromatin domains move similarly, but PC bodies move in a larger volume than chromatin domains. To explain this difference, one might argue that condensed chromatin domains would form much larger structures than PC bodies. This is difficult to ascertain until the identity of these DAPI- and histone-dense regions is better understood. Genome-wide analysis of chromatin components has recently identified five different types of chromatin in *Drosophila* cells, among which three contained silent genes [55]. In addition to heterochromatin and Polycomb-repressed chromatin, a third type of silent chromatin was uncovered, which is composed of very large genomic domains encompassing half of the genomic euchromatin. We propose that this silent chromatin portion of the genome is physically manifested as the DAPI- and histone-dense chromatin that we identified to be distinct from PC bodies.

## Materials and Methods

### Fly lines

Flies were raised in standard cornmeal yeast medium. Oregon R w^1118^ was used for I-FISH. The fly lines expressing PH-GFP or PC-GFP has been previously described [33], [34]. The fly line BL#5941 of the Bloomington Drosophila Stock Centre expresses H2Av-GFP. The fly line containing a UAS-mH2B-RFP transgene on chromosome 3 was previously characterized [56]. To induce ubiquitous expression of H2B-mRFP, we used the fly line BL #5138 containing Gal4-Tub.

### Immuno-FISH and immuno-localization

A detailed protocol of the I-FISH is available at http://www.epigenome-noe.net/WWW/researchtools/protocol.php?protid=5. The probes used in FISH are listed in Table 1. We always focused on epidermal cells in the anterior part of the embryos. Immuno-FISH against PC was performed with a PC rabbit polyclonal antibody developed in our lab [15]. To compare the localization of Histone H3K27me3 and the distribution of PC-GFP, we used staged embryos which were mechanically broken and then immediately fixed by using 4% of PFA in PBS. After permeabilization with 0.2% of Triton X100 during 15 minutes and blocking with 2% BSA in PBS for 30 minutes, a rabbit polyclonal antibody (Upstate Biotechnology, #07-449) was used to detect Histone H3K27me3. The same protocol was used to compare the nuclear distribution of PC-GFP and PH-GFP with endogenous PC and PH proteins, by using a PC rabbit polyclonal antibody and a previously described PH goat polyclonal antibody [15].

**Table 1:** Primers and genomic fragments used for the FISH probes.

Locus	Genomic fragments used for the FISH probes	Primers of the PCR fragments Sequence 5′ – 3′ Forward (F)	Primers of the PCR fragments Sequence 5′ – 3′ Reverse (R)
***Antp***	antp 1	F : ggggactgaatggatggg	R : ctctatatttgcatagcttgag
	antp2	F : ggcaaacaaaggggttgc	R : ggtggtagtggtaggttg
	antp4	F : gtgcgtgccagccttcag	R : gaatacacccaagagacatttc
	antp5	F : taataacacgttccccagac	R : taacatactcgcagaaccc
	antp6	F : ctacgtgttggcattagact	R : acctcttccttctcgacc
	antp7	F : taaggcagtataataaaaagtg	R : caatctgctttcagatctgcat
	antp9	F : aggctgaaagtaaaagccag	R : gtttcgattccactgcgg
	antp10	F : gtggcagcgcagtatataa	R : gcaaacagaaactttcgacc
	antp11	F : cacatcagaaaagcattcagac	R : tacacagttgcctgggtc
***Fab-7 (Abd-B)***	fab1	F : ttggcaaataacgacttatagc	R : ggcgcagatacatttgtatctt
	fab3	F : aaatcgacccttgttgtcc	R : cgccaaccagcacaaaca
	fab4	F : ttcatttgtgtaaaacaagagg	R : ttcagagaatatcaaactgccc
	fab5	F : caccattaaaaggcgacaac	R : ctcaatacttcaacacacattc
	fab6	F : ccacattcagcgactacg	R :ccagaggggaaacaacaaaa
	fab7	F : gaaatagctccaccgtgc	R : gcacagctgcgaatggcg
	fab8	F : caccgcagttggtagtttta	R : tgatccctgggctcctcc
	fab9	F : cagcatccaacaagctcc	R : agcggcataggccgaatg
***lbl/lbe***	lbl1	F : ttcgtgtagccaaccctct	R : aatagctgcacagcgtttca
	lbl2	F : gagcaccatttgtggatgtg	R : cagctgatttgatcggttga
	lbl3	F : gagaaatcaacgccgctaag	R : ttggagctaagccgtaagga
	lbl4	F : ttggcttcgactcctcatct	R : acagggcgtgtctcgtctat
	lbl5	F : tcattgccgttgtgtcaaat	R : tatggggcccacttaatacg
	lbl6	F : caacgagcggcacatagata	R : ctcgaaacttggtcgaaagc
***hh***	hh1	F : cgctttgggtgtcgtat	R : agtgtttgaactgcacgtaa
	hh2	F : gatgaggtgtggatctgtag	R : gccgggaatcaaaggata
	hh3	F : ttaccacttcgaaagatggtatag	R : tgaatgccacgggattag
	hh4	F : tagtcaatcgcccgaagga	R : gacggctgaacggtaga
	hh5	F : gtgtgtttgacctccatacc	R : ccgaagactcaggcacta
***svp***	svp1	F : gtggccaactcaacctgtct	R : gggccataggcattacttga
	svp2	F : tctgggagctgttccgtagt	R : atcaccagcatcccaggtag
	svp3	F : gttggccgcttttattgtgt	R : acatggatctgctcccattc
	svp4	F : gagtgcgaggcacactaaca	R : gcagtggcactcgatactga
	svp6	F : gggatgcagctggaatgtat	R : ggagtggtgtggagtggagt
***hth***	hth1	F : tttcggcgatctttgatttc	R : ggaaactcgcaaaacgagag
	hth2	F : ccataactcagcgagtgcaa	R : tgaccagcgaaaacagacac
	hth3	F : gcaaataaagcggacagctc	R : gtaggcggtaatcggaatca
	hth4	F : cccgctaatctcaaggacac	R : ctggaaaatgacccatgctt
	hth5	F : tttgggtcacagtttgtgga	R : gtggctttttgctcgagagt
	hth6	F : atgccgcttgcctttactta	R : cacccagtaaggcccataga
***beat-Vc***	bvc1	F : aaacgaggctaaacgagcaa	R : tcctctttgggcagaacaat
	bvc2	F : cctaagtgcgcattatgcaa	R : ataaatggggtggagtgcag
	bvc3	F : tgacacctttaacggggaac	R : ttaacggtttacgggctttg
	bvc4	F : acatttcgcaactcccaatc	R : gggtcgagcatttgattcat
	bvc5	F : tattgccacgacaactacgg	R : cgtccggcaatcaagtaaat
	bvc6	F : ttacgccctaaatggaatgc	R : tgcctttagatccagcgact

### Time-lapse microscopy

Embryos were manually dechorionated, recovered by oil voltalef 3S and mounted between two cover-slips on a slide. Images were collected by using a Zeiss LSM 510 Meta and a 60X N.A. 1.4 objective. Although complete mitosis still occurs in embryos which stayed more than 30 min under the microscope, embryos were not kept more than 30 min under the microscope for time-lapse experiments. Images of PC-GFP have been acquired with a pixel size of 70 nm, whereas the motion of H2Av-GFP chromatin domains and I-FISH experiments were monitored with images having pixels of 48 nm. Short time-lapse experiments were acquired by collecting 2D images (512*128 pixels) every 250 ms for 15 s. For long time-lapse experiments, we collected 1 volume (512*100 pixels: 6 optical sections with a Z-step of 500 nm) every 3 s during 3 min. We looked at epidermal cells in the anterior part of the embryos. A median (3*3) filter was applied to reduce the noise within images of both PC-GFP and H2Av-GFP. The time-lapse experiments performed on embryos expressing PH-GFP and PC-GFP in Figure 6 were collected by using a Zeiss LSM 780 with GaAsP detector. A pixel size of 66 nm was used to acquire 2D images (512*256 pixels) every 250 ms for 15 s. Long 4D tracking experiments were recorded with a pixel size of 88 nm and by acquiring one volume of 12 sections (512*512 pixels) with a z-step of 0.5 µm every 10 sec during 30 min.

Images of H2Av-GFP and PC-GFP were segmented using the volocity software (Improvision) and we used the centre of mass of segmented chromatin domains and PC bodies to extract their tracks. To monitor the global displacement of cell nuclei, we used the H2Av-GFP or PC-GFP signal to compute their centre of mass during the entire time-lapse experiment. For long time-lapse experiments, 4D movies are required because PC bodies also move in the Z-axis and shift out of focus. However, the Z-step of 500 nm is high compared to the 70 nm lateral size of pixels. Therefore, to avoid biases induced by under-sampling along the Z-axis, calculations applied to 4D images were performed without taking into account Z displacements. To calculate the MSD or visualize tracks, we used the relative position of chromatin domain or PC body compared to the centre of the nucleus. The MSD measures the average square distance between the positions of one PC body or one chromatin domain between two time-points. In contrast, the MSC measures the mean square change in distance between two PC bodies or chromatin domains occurring between two time-points. Therefore, we did not use the relative position of PC bodies and chromatin domains to calculate MSC. Time-lapse experiments comparing the motion of PC bodies (measured by using either PC-GFP or PH-GFP) with that of H2av-GFP chromatin domains (Figure 6) were analyzed using the software Bitplane (Imaris). A Gaussian filter was applied to images in order to decrease their noise and tracks of PC bodies and chromatin domains were computed using the options “spots” and “tracking spots”.

To characterize the distribution of MSD and MSC of both chromatin domains and PC bodies, we used the Kolmogorov-Smirnov test (http://www.physics.csbsju.edu/stats/KS-test.n.plot_form.html), which indicated that they have Log-normal distributions. Therefore, we did not show standard deviations, and performed statistical tests by using t-test on the log values of MSD and MSC. We calculated the p-values for correlation coefficients by using a website (http://www.danielsoper.com/statcalc/calc44.aspx).

### Half-bleach and FLIP

Nuclei of embryos expressing H2Av-GFP were half bleached 0.3 s after the beginning of 2D time-lapse experiments for 10.5 s. Then, 3D time-series were recorded for 3 min by collecting 3 µm-thick volumes every 3 s. After a (3*3) median filter, 2D and 3D images movies were visualized with the volocity software. FLIP experiments were performed on embryos expressing PC-GFP by collecting 2D images every 1.3 s for 80 s. A fixed spot of about 500 nm was bleached for 0.3 s every 2 images during the entire time-lapse experiments. Measurements of average and local maximum intensities of PC-GFP during FLIP experiments were done using ImageJ. We looked at epidermal cells in the anterior part of the embryos in both half-bleach and FLIP experiments.

## Supporting Information

Figure S1 Both PC-GFP and PH-GFP accumulate in PC bodies. A: 3D visualization of PC-GFP compared to immuno-labeling performed with specific antibodies against PH and H3K27me3. Profile showing that local accumulations of PC-GFP co-localize with H3K27me3 and PH. B: 3D visualization of PH-GFP compared to immuno-labeling performed with specific antibodies against PC. Profile indicating that local accumulations of PH-GFP co-localize with PC. H2av-GFP co-localizes with DAPI staining and accumulates in nuclear domains which do not contain H3K27me3. C: 3D visualization of an immuno-labeling performed with specific antibody against H3K27me3 compared to DAPI staining. Profile showing that H3K27me3 does not correlate with DAPI staining. D: 3D visualization of H2av-GFP compared to DAPI staining. Profile indicating that both DAPI and H2av-GFP co-localize within euchromatin.
(TIF)

Figure S2 PC enrichment within PC bodies during fly's embryogenesis. A: Typical examples of nuclei stained with DAPI (blue), immuno-labeled with a polyclonal antibody against PC (green) and FISH performed with probes located in ANT-C, BX-C, NK-C, hh, hth, svp and beat-Vc (red), taken from embryos at stages 6–7, 11 and 15. Bars measure 2 µm. B–D: Cumulative histograms of PC enrichment measured within the FISH volumes at developmental stages 6–7 (C), 11 (D) and 15 (E).
(TIF)

Figure S3 Motion of PC bodies during embryonic development. A–C: Example of 2D images taken from 15 s movies of embryos expressing PC-GFP at stages 5(A), 11(B) and 15(C). For example, a weak PC body (arrows) obviously moves compared to another intense PC body. D–F: Example of 2D images taken from 15 s movies of embryos expressing PH-GFP at stages 5(D), 11(E) and 15(F). Bars measure 2 µm.
(TIF)

Figure S4 Long Time-lapse imaging of PC bodies. A: 4D images of embryos expressing PH-GFP at stage 11 illustrating that one intense PC body is composed of several weaker ones (arrows). B: 4D images of embryos expressing PH-GFP at stage 5 showing a rapid dissociation of one intense PC bodies in two distinct ones (arrows). C: 4D images of embryos expressing PH-GFP at stage 11 monitoring a stable association of two PC bodies (arrows). D: 4D images of embryos expressing PC-GFP at stage 11 illustrating the increase of fluorescence observed after association of two PC bodies (arrows). E: 4D images of embryo expressing PC-GFP at stage 11, showing the decrease of fluorescence observed after dissociation of one PC body (arrows). Bars measure 2 µm.
(TIF)

Figure S5 Motion of chromatin domains during embryonic development. A–C: Example of 2D images taken from 15 s movies of embryos expressing H2Av-GFP at stages 5 (A), 11 (B) and 15 (C). D–F: Cell nuclei expressing H2Av-GFP were half-bleached and subsequent time-lapse movies were collected at stages 5 (D), 11 (E) and 15 (F). During the entire time-lapse experiments, the borderline between bleached and unbleached areas stays clearly visible, whereas motion of distinct chromatin domains is easily observable (arrows in D). Obvious coordinated motions of several chromatin domains are also seen (arrows in E and braces in F). Bars measure 2 µm.
(TIF)

Figure S6 Complex motion of PC bodies during embryonic development. A: MSD curves characterizing the motion of PC bodies during embryogenesis. The results obtained with projections along the Z-axis of long 4D tracking fit with the data found with fast 2-D time-lapse experiments (compare the red, green and blue curves with their corresponding grey, yellow and black ones). B: Tables showing the average radius (in nm) of the volumes in which PC bodies move. C–E: Scatter-plots between MSD of each PC body and its corresponding MSC computed for motions of 1 s (E), 5 s (F) or 60 s (G).
(TIF)

Figure S7 Complex motion of chromatin domains during embryonic development. A: MSD curves characterizing the motion of chromatin domains during embryogenesis. B: Tables showing the average radius (in nm) of the volumes in which chromatin domains move. C–D: Scatter-plots between the MSD of each chromatin domain and its corresponding MSC computed for motions of 1 s (E) or 5 s (F).
(TIF)

Figure S8 Effect of PC-GFP or H2Av-GFP enrichment on the motions of PC bodies or chromatin domains. A–C: MSD curves comparing the average motion between the most intense and the weakest PC body tracked within one nucleus at stages 5 (A), 11 (B) and 15 (C). D–E: Scatter-plots between the MSD/t reached after 1 s of PC bodies and chromatin domains and their respective enrichments in PC-GFP (D) or H2Av-GFP (E). Full squares and diamonds point tracks showing only constrained motion, whereas empty squares and diamonds correspond to tracks displaying both constrained and long-range motions. F–G: Tables presenting the coefficient of correlation (r) calculated between enrichments of PC-GFP (F) or H2Av-GFP (G) and the motions of PC bodies and chromatin domains (c = tracks only showing constrained motion; LR = tracks displaying both constrained and long-range motions; Total = c+LR). Colors depends on the p-value associated to the coefficient of correlation calculated for both normal and lognormal distributions of MSD/t (Red p<0.001; Orange p<0.01; yellow p<0.05 and grey p>0.05).
(TIF)

Figure S9 The motions of chromatin domains and PC bodies as well as the PC-GFP kinetics depend on temperature. A–F: Decrease of temperature slows down motions of chromatin domains and PC bodies. MSD/t curves quantifying the motions of chromatin domains (A–C) or PC bodies (D–F) at both 18°C and 25°C, in embryos at stages 5 (A and D), 11 (B and E) and 15 (C and F). G–I: Temperature affects kinetics of PC-GFP. Curves monitoring the loss of fluorescence occurring during FLIP experiments performed at both 18°C and 25°C in embryos at stages 5 (G), 11 (H) and 15 (I). J: Table of p-values comparing the MSD/t of chromatin domains and PC bodies reached after 5 s at 18°C with the ones calculated at 25°C. K: Table of p-values comparing the loss of fluorescence inside PC bodies and within the nucleoplasm at 18°C with the ones measured at 25°C. (J and K: red p<0.001; orange p<0.01; yellow p<0.05 and grey p>0.05).
(TIF)

Figure S10 Effect of temperature on the motion of PC bodies and chromatin domains during embryogenesis. A–F: MSD/t curves quantifying constrained (A–C) and long-range (D–F) motions of chromatin domains at 18°C and 25°C, in embryos at stages 5 (A and D), 11 (B and E) and 15 (C and F). G–L: MSD/t curves quantifying constrained (G–I) and long-range (J–L) motions of PC bodies at 18°C and 25°C, in embryos at stages 5 (G and J), 11 (H and K) and 15 (I and L). M–N: Tables of p-values comparing the motions of chromatin domains (M) or PC bodies (N) measured at 18°C with the ones observed at 25°C.
(TIF)

Video S1 Fast motion of PC bodies: 15 s movies of embryos expressing PC-GFP at stages 5, 11 and 15.
(WMV)

Video S2 Slow motion of PC bodies: 3 min movies of embryos expressing PC-GFP at stages 5, 11 and 15.
(WMV)

Video S3 Fast motion of chromatin domains: 15 s movies of embryos expressing H2av-GFP at stages 5, 11 and 15.
(WMV)

Video S4 Coordinated motion of chromatin domains: cell nuclei expressing H2Av-GFP were half-bleached and subsequent time-lapse movies show obvious coordinated motions of several chromatin domains.
(WMV)

Video S5 Chromatin domains and PC bodies form distinct structures undergoing occasional coordinated long-range motion: movies of embryos at developmental stage 15 expressing both H2B-RFP and PC-GFP.
(WMV)

Video S6 Long Time-lapse imaging of PC bodies: 30 and 10 minutes movies of embryos expressing PC-GFP at stages 5, 11 and 15.
(WMV)

Video S7 Long Time-lapse imaging of PC bodies: 30 and 10 minutes movies of embryos expressing PH-GFP at stages 5, 11 and 15.
(WMV)

Video S8 Fast motion of PC bodies: 15 s movies of embryos expressing PH-GFP at stages 5, 11 and 15.
(WMV)

## References

[1] Jenuwein T, Allis CD (2001). Translating the histone code.. Science.

[2] Shao Z, Raible F, Mollaaghababa R, Guyon JR, Wu CT (1999). Stabilization of chromatin structure by PRC1, a Polycomb complex.. Cell.

[3] Simon JA, Kingston RE (2009). Mechanisms of Polycomb gene silencing: knowns and unknowns.. Nat Rev Mol Cell Biol.

[4] Schwartz YB, Pirrotta V (2007). Polycomb silencing mechanisms and the management of genomic programmes.. Nat Rev Genet.

[5] Schuettengruber B, Chourrout D, Vervoort M, Leblanc B, Cavalli G (2007). Genome regulation by polycomb and trithorax proteins.. Cell.

[6] Negre N, Hennetin J, Sun LV, Lavrov S, Bellis M (2006). Chromosomal distribution of PcG proteins during Drosophila development.. PLoS Biol.

[7] Schuettengruber B, Ganapathi M, Leblanc B, Portoso M, Jaschek R (2009). Functional anatomy of polycomb and trithorax chromatin landscapes in Drosophila embryos.. PLoS Biol.

[8] Muller J, Verrijzer P (2009). Biochemical mechanisms of gene regulation by polycomb group protein complexes.. Curr Opin Genet Dev.

[9] Lanzuolo C, Roure V, Dekker J, Bantignies F, Orlando V (2007). Polycomb response elements mediate the formation of chromosome higher-order structures in the bithorax complex.. Nat Cell Biol.

[10] Bantignies F, Roure V, Comet I, Leblanc B, Schuettengruber B (2011). Polycomb-dependent regulatory contacts between distant Hox loci in Drosophila.. Cell.

[11] Tolhuis B, Blom M, Kerkhoven RM, Pagie L, Teunissen H (2011). Interactions among Polycomb Domains Are Guided by Chromosome Architecture.. PLoS Genet.

[12] Bantignies F, Grimaud C, Lavrov S, Gabut M, Cavalli G (2003). Inheritance of Polycomb-dependent chromosomal interactions in Drosophila.. Genes Dev.

[13] Vazquez J, Muller M, Pirrotta V, Sedat JW (2006). The Mcp element mediates stable long-range chromosome-chromosome interactions in Drosophila.. Mol Biol Cell.

[14] Li HB, Muller M, Bahechar IA, Kyrchanova O, Ohno K (2010). Insulators, not Polycomb Response Elements, are required for long-range interactions between Polycomb targets in Drosophila.. Mol Cell Biol.

[15] Grimaud C, Bantignies F, Pal-Bhadra M, Ghana P, Bhadra U (2006). RNAi components are required for nuclear clustering of Polycomb group response elements.. Cell.

[16] Buchenau P, Hodgson J, Strutt H, Arndt-Jovin DJ (1998). The distribution of polycomb-group proteins during cell division and development in Drosophila embryos: impact on models for silencing.. J Cell Biol.

[17] Messmer S, Franke A, Paro R (1992). Analysis of the functional role of the Polycomb chromo domain in Drosophila melanogaster.. Genes Dev.

[18] Eskeland R, Leeb M, Grimes GR, Kress C, Boyle S (2010). Ring1B compacts chromatin structure and represses gene expression independent of histone ubiquitination.. Mol Cell.

[19] Terranova R, Yokobayashi S, Stadler MB, Otte AP, van Lohuizen M (2008). Polycomb group proteins Ezh2 and Rnf2 direct genomic contraction and imprinted repression in early mouse embryos.. Dev Cell.

[20] Ficz G, Heintzmann R, Arndt-Jovin DJ (2005). Polycomb group protein complexes exchange rapidly in living Drosophila.. Development.

[21] Ren X, Vincenz C, Kerppola TK (2008). Changes in the distributions and dynamics of polycomb repressive complexes during embryonic stem cell differentiation.. Mol Cell Biol.

[22] Marshall WF, Straight A, Marko JF, Swedlow J, Dernburg A (1997). Interphase chromosomes undergo constrained diffusional motion in living cells.. Curr Biol.

[23] Vazquez J, Belmont AS, Sedat JW (2001). Multiple regimes of constrained chromosome motion are regulated in the interphase Drosophila nucleus.. Curr Biol.

[24] Chubb JR, Boyle S, Perry P, Bickmore WA (2002). Chromatin motion is constrained by association with nuclear compartments in human cells.. Curr Biol.

[25] Heun P, Laroche T, Shimada K, Furrer P, Gasser SM (2001). Chromosome dynamics in the yeast interphase nucleus.. Science.

[26] Thakar R, Csink AK (2005). Changing chromatin dynamics and nuclear organization during differentiation in Drosophila larval tissue.. J Cell Sci.

[27] Chuang CH, Carpenter AE, Fuchsova B, Johnson T, de Lanerolle P (2006). Long-range directional movement of an interphase chromosome site.. Curr Biol.

[28] Dundr M, Ospina JK, Sung MH, John S, Upender M (2007). Actin-dependent intranuclear repositioning of an active gene locus in vivo.. J Cell Biol.

[29] Platani M, Goldberg I, Lamond AI, Swedlow JR (2002). Cajal body dynamics and association with chromatin are ATP-dependent.. Nat Cell Biol.

[30] Gorisch SM, Wachsmuth M, Ittrich C, Bacher CP, Rippe K (2004). Nuclear body movement is determined by chromatin accessibility and dynamics.. Proc Natl Acad Sci U S A.

[31] Alkema MJ, Bronk M, Verhoeven E, Otte A, van 't Veer LJ (1997). Identification of Bmi1-interacting proteins as constituents of a multimeric mammalian polycomb complex.. Genes Dev.

[32] Saurin AJ, Shiels C, Williamson J, Satijn DP, Otte AP (1998). The human polycomb group complex associates with pericentromeric heterochromatin to form a novel nuclear domain.. J Cell Biol.

[33] Netter S, Faucheux M, Theodore L (2001). Developmental dynamics of a polyhomeotic-EGFP fusion in vivo.. DNA Cell Biol.

[34] Dietzel S, Niemann H, Bruckner B, Maurange C, Paro R (1999). The nuclear distribution of Polycomb during Drosophila melanogaster development shown with a GFP fusion protein.. Chromosoma.

[35] Schwartz YB, Kahn TG, Nix DA, Li XY, Bourgon R (2006). Genome-wide analysis of Polycomb targets in Drosophila melanogaster.. Nat Genet.

[36] Tolhuis B, de Wit E, Muijrers I, Teunissen H, Talhout W (2006). Genome-wide profiling of PRC1 and PRC2 Polycomb chromatin binding in Drosophila melanogaster.. Nat Genet.

[37] Manak JR, Dike S, Sementchenko V, Kapranov P, Biemar F (2006). Biological function of unannotated transcription during the early development of Drosophila melanogaster.. Nat Genet.

[38] Kosman D, Mizutani CM, Lemons D, Cox WG, McGinnis W (2004). Multiplex detection of RNA expression in Drosophila embryos.. Science.

[39] Jagla K, Jagla T, Heitzler P, Dretzen G, Bellard F (1997). ladybird, a tandem of homeobox genes that maintain late wingless expression in terminal and dorsal epidermis of the Drosophila embryo.. Development.

[40] Rieckhof GE, Casares F, Ryoo HD, Abu-Shaar M, Mann RS (1997). Nuclear translocation of extradenticle requires homothorax, which encodes an extradenticle-related homeodomain protein.. Cell.

[41] Kerber B, Fellert S, Hoch M (1998). Seven-up, the Drosophila homolog of the COUP-TF orphan receptors, controls cell proliferation in the insect kidney.. Genes & development.

[42] Swaminathan J, Baxter EM, Corces VG (2005). The role of histone H2Av variant replacement and histone H4 acetylation in the establishment of Drosophila heterochromatin.. Genes Dev.

[43] Mavrich TN, Jiang C, Ioshikhes IP, Li X, Venters BJ (2008). Nucleosome organization in the Drosophila genome.. Nature.

[44] Leach TJ, Mazzeo M, Chotkowski HL, Madigan JP, Wotring MG (2000). Histone H2A.Z is widely but nonrandomly distributed in chromosomes of Drosophila melanogaster.. J Biol Chem.

[45] Cmarko D, Verschure PJ, Otte AP, van Driel R, Fakan S (2003). Polycomb group gene silencing proteins are concentrated in the perichromatin compartment of the mammalian nucleus.. J Cell Sci.

[46] Francis NJ, Kingston RE, Woodcock CL (2004). Chromatin compaction by a polycomb group protein complex.. Science.

[47] Margueron R, Li G, Sarma K, Blais A, Zavadil J (2008). Ezh1 and Ezh2 maintain repressive chromatin through different mechanisms.. Mol Cell.

[48] Fitzgerald DP, Bender W (2001). Polycomb group repression reduces DNA accessibility.. Mol Cell Biol.

[49] Levi V, Ruan Q, Plutz M, Belmont AS, Gratton E (2005). Chromatin dynamics in interphase cells revealed by tracking in a two-photon excitation microscope.. Biophys J.

[50] Simonis M, Klous P, Splinter E, Moshkin Y, Willemsen R (2006). Nuclear organization of active and inactive chromatin domains uncovered by chromosome conformation capture-on-chip (4C).. Nat Genet.

[51] Wachsmuth M, Caudron-Herger M, Rippe K (2008). Genome organization: balancing stability and plasticity.. Biochim Biophys Acta.

[52] Cremer T, Cremer M, Dietzel S, Muller S, Solovei I (2006). Chromosome territories–a functional nuclear landscape.. Curr Opin Cell Biol.

[53] Thakar R, Gordon G, Csink AK (2006). Dynamics and anchoring of heterochromatic loci during development.. J Cell Sci.

[54] Misteli T (2001). Protein dynamics: implications for nuclear architecture and gene expression.. Science.

[55] Filion GJ, van Bemmel JG, Braunschweig U, Talhout W, Kind J (2010). Systematic protein location mapping reveals five principal chromatin types in Drosophila cells.. Cell.

[56] Langevin J, Le Borgne R, Rosenfeld F, Gho M, Schweisguth F (2005). Lethal giant larvae controls the localization of notch-signaling regulators numb, neuralized, and Sanpodo in Drosophila sensory-organ precursor cells.. Curr Biol.

