# Supplementary figures and images for: Progressive Polycomb Assembly on H3K27me3 Compartments Generates Polycomb Bodies with Developmentally Regulated Motion
Source: PLoS Genet. 2012 Jan 19;8(1):e1002465. doi: 10.1371/journal.pgen.1002465 (PMC3262012; doi:10.1371/journal.pgen.1002465)

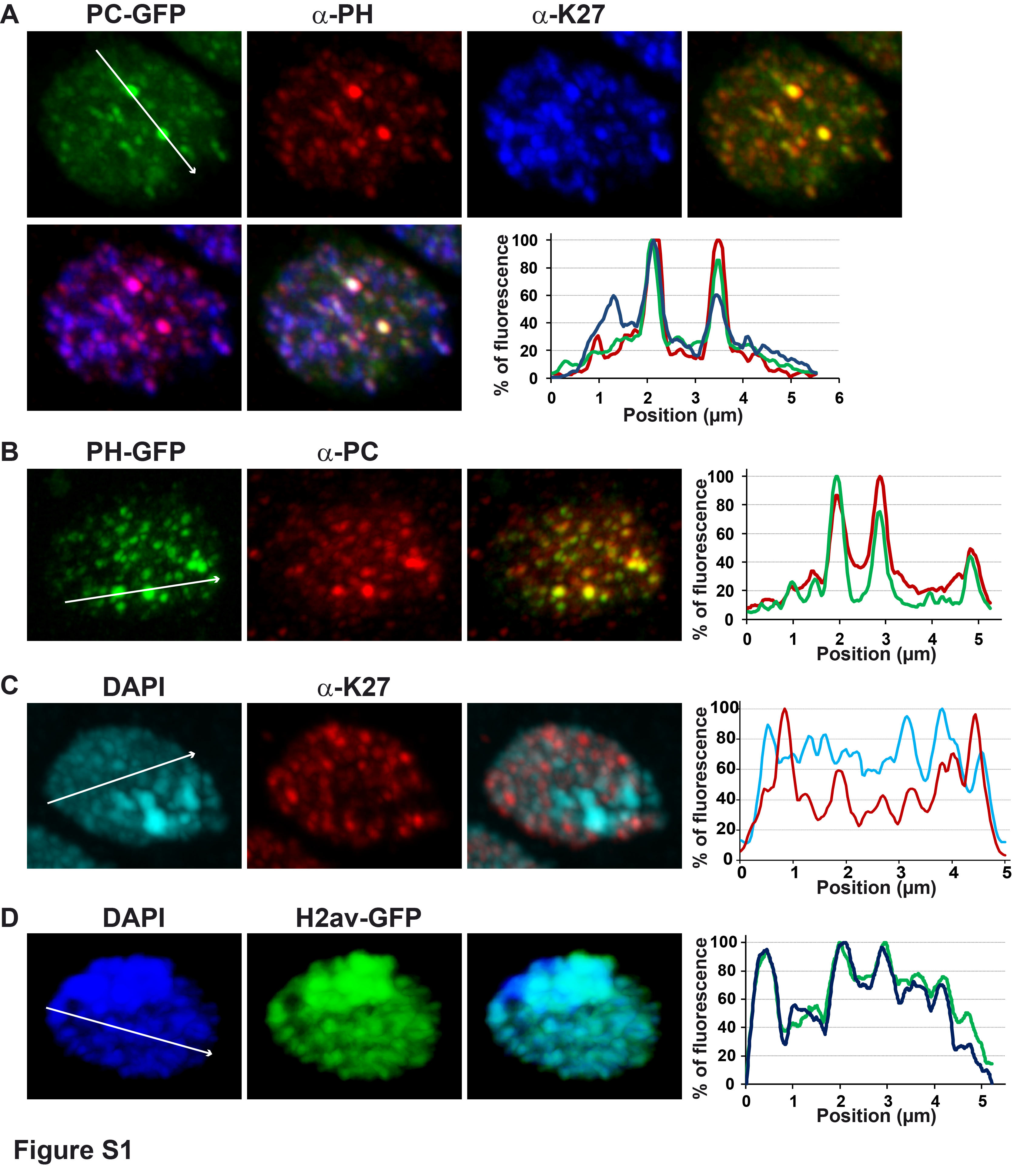

Supplement: Figure S1 — Both PC-GFP and PH-GFP accumulate in PC bodies. A: 3D visualization of PC-GFP compared to immuno-labeling performed with specific antibodies against PH and H3K27me3. Profile showing that local accumulations of PC-GFP co-localize with H3K27me3 and PH. B: 3D visualization of PH-GFP compared to immuno-labeling performed with specific antibodies against PC. Profile indicating that local accumulations of PH-GFP co-localize with PC. H2av-GFP co-localizes with DAPI staining and accumulates in nuclear domains which do not contain H3K27me3. C: 3D visualization of an immuno-labeling performed with specific antibody against H3K27me3 compared to DAPI staining. Profile showing that H3K27me3 does not correlate with DAPI staining. D: 3D visualization of H2av-GFP compared to DAPI staining. Profile indicating that both DAPI and H2av-GFP co-localize within euchromatin. (TIF) [file pgen.1002465.s001.tif]

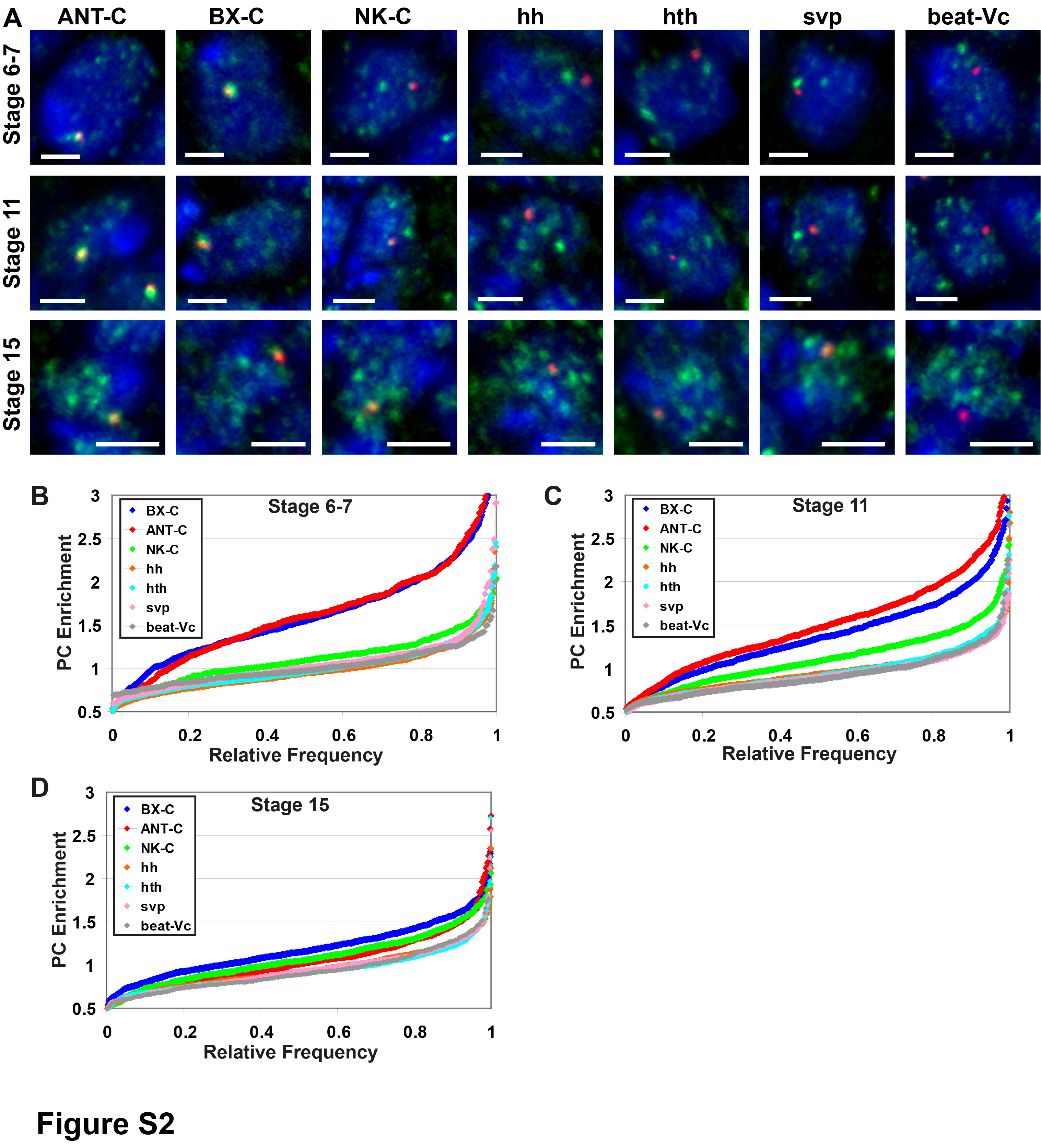

Supplement: Figure S2 — PC enrichment within PC bodies during fly's embryogenesis. A: Typical examples of nuclei stained with DAPI (blue), immuno-labeled with a polyclonal antibody against PC (green) and FISH performed with probes located in ANT-C, BX-C, NK-C, hh, hth, svp and beat-Vc (red), taken from embryos at stages 6–7, 11 and 15. Bars measure 2 µm. B–D: Cumulative histograms of PC enrichment measured within the FISH volumes at developmental stages 6–7 (C), 11 (D) and 15 (E). (TIF) [file pgen.1002465.s002.tif]

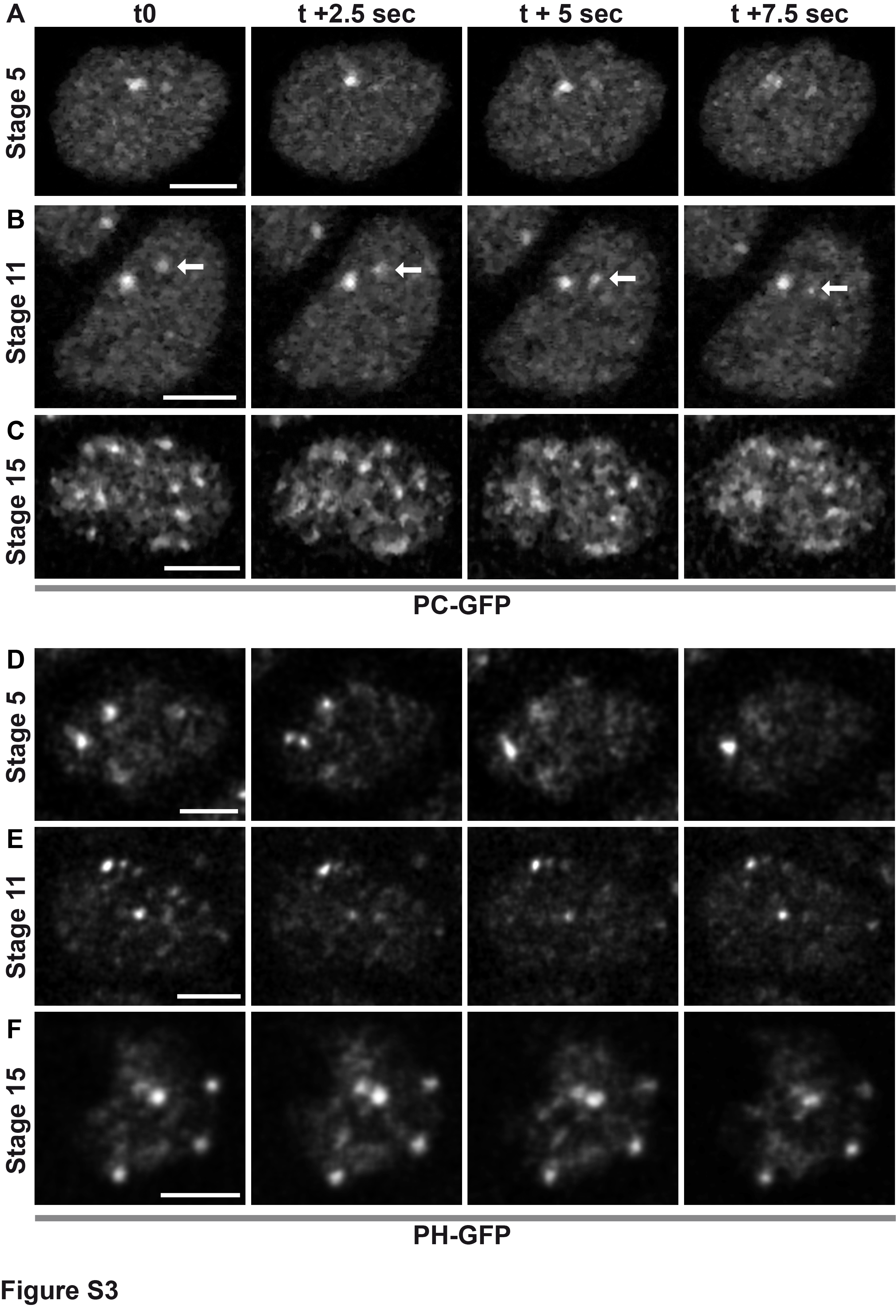

Supplement: Figure S3 — Motion of PC bodies during embryonic development. A–C: Example of 2D images taken from 15 s movies of embryos expressing PC-GFP at stages 5(A), 11(B) and 15(C). For example, a weak PC body (arrows) obviously moves compared to another intense PC body. D–F: Example of 2D images taken from 15 s movies of embryos expressing PH-GFP at stages 5(D), 11(E) and 15(F). Bars measure 2 µm. (TIF) [file pgen.1002465.s003.tif]

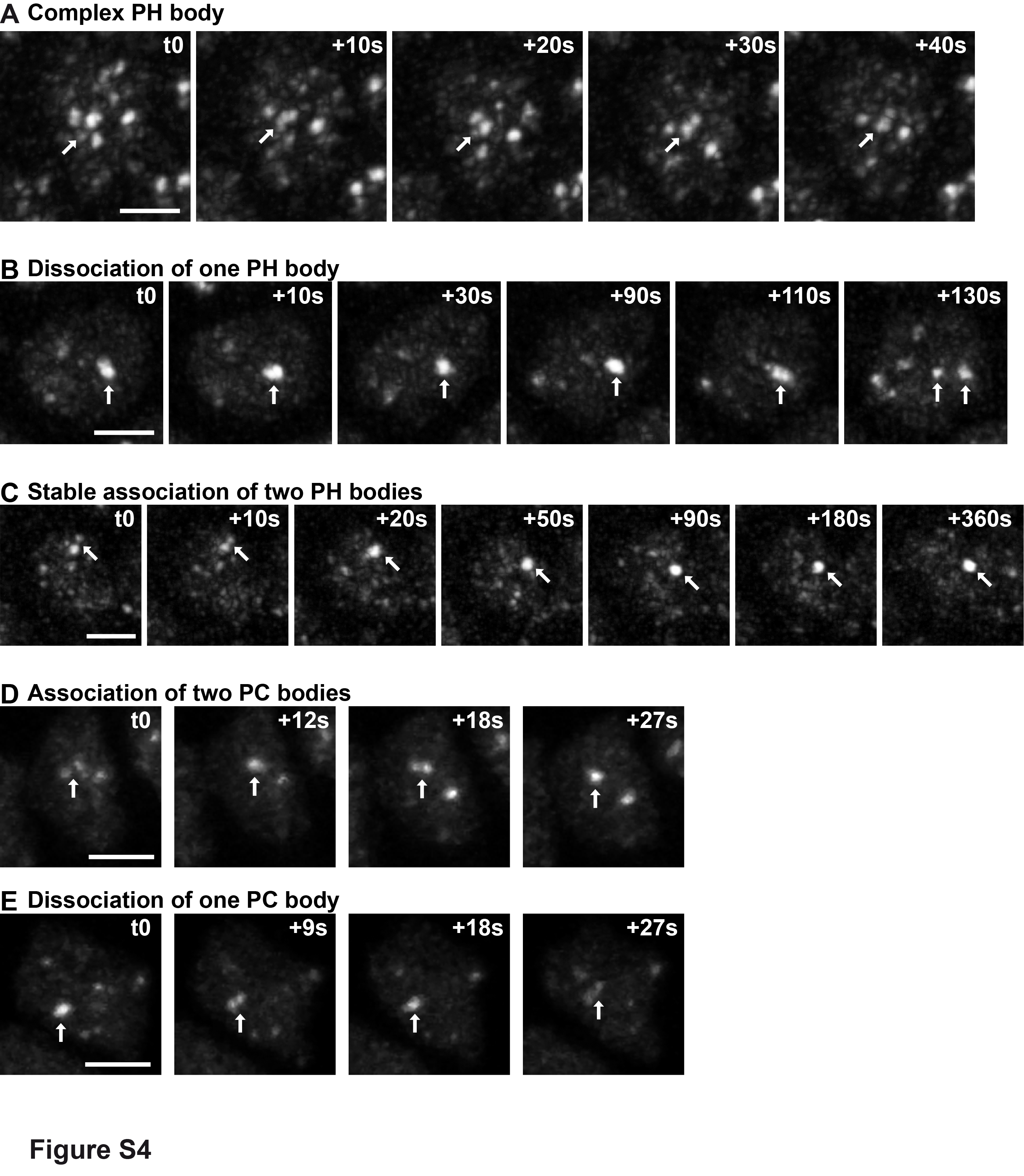

Supplement: Figure S4 — Long Time-lapse imaging of PC bodies. A: 4D images of embryos expressing PH-GFP at stage 11 illustrating that one intense PC body is composed of several weaker ones (arrows). B: 4D images of embryos expressing PH-GFP at stage 5 showing a rapid dissociation of one intense PC bodies in two distinct ones (arrows). C: 4D images of embryos expressing PH-GFP at stage 11 monitoring a stable association of two PC bodies (arrows). D: 4D images of embryos expressing PC-GFP at stage 11 illustrating the increase of fluorescence observed after association of two PC bodies (arrows). E: 4D images of embryo expressing PC-GFP at stage 11, showing the decrease of fluorescence observed after dissociation of one PC body (arrows). Bars measure 2 µm. (TIF) [file pgen.1002465.s004.tif]

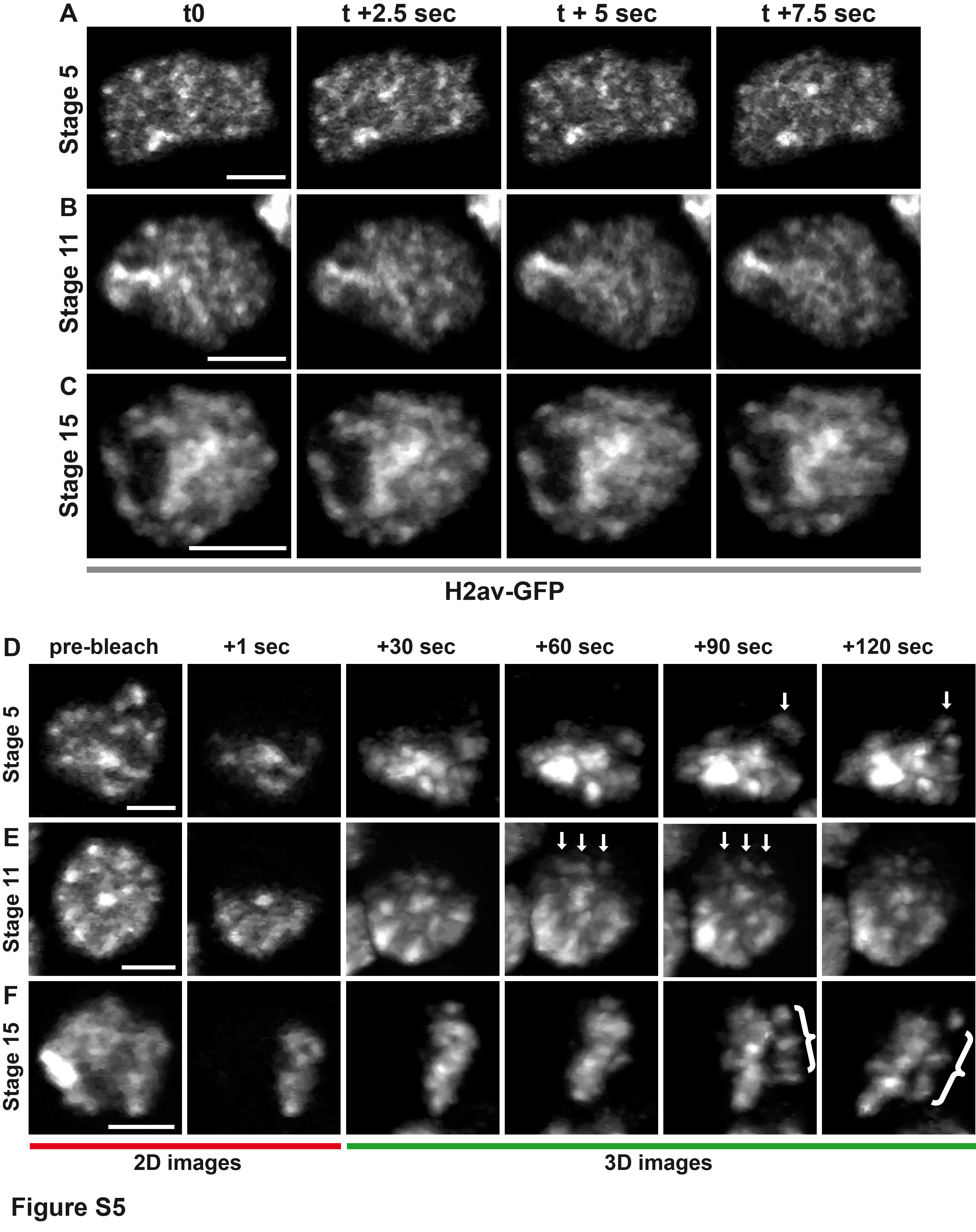

Supplement: Figure S5 — Motion of chromatin domains during embryonic development. A–C: Example of 2D images taken from 15 s movies of embryos expressing H2Av-GFP at stages 5 (A), 11 (B) and 15 (C). D–F: Cell nuclei expressing H2Av-GFP were half-bleached and subsequent time-lapse movies were collected at stages 5 (D), 11 (E) and 15 (F). During the entire time-lapse experiments, the borderline between bleached and unbleached areas stays clearly visible, whereas motion of distinct chromatin domains is easily observable (arrows in D). Obvious coordinated motions of several chromatin domains are also seen (arrows in E and braces in F). Bars measure 2 µm. (TIF) [file pgen.1002465.s005.tif]

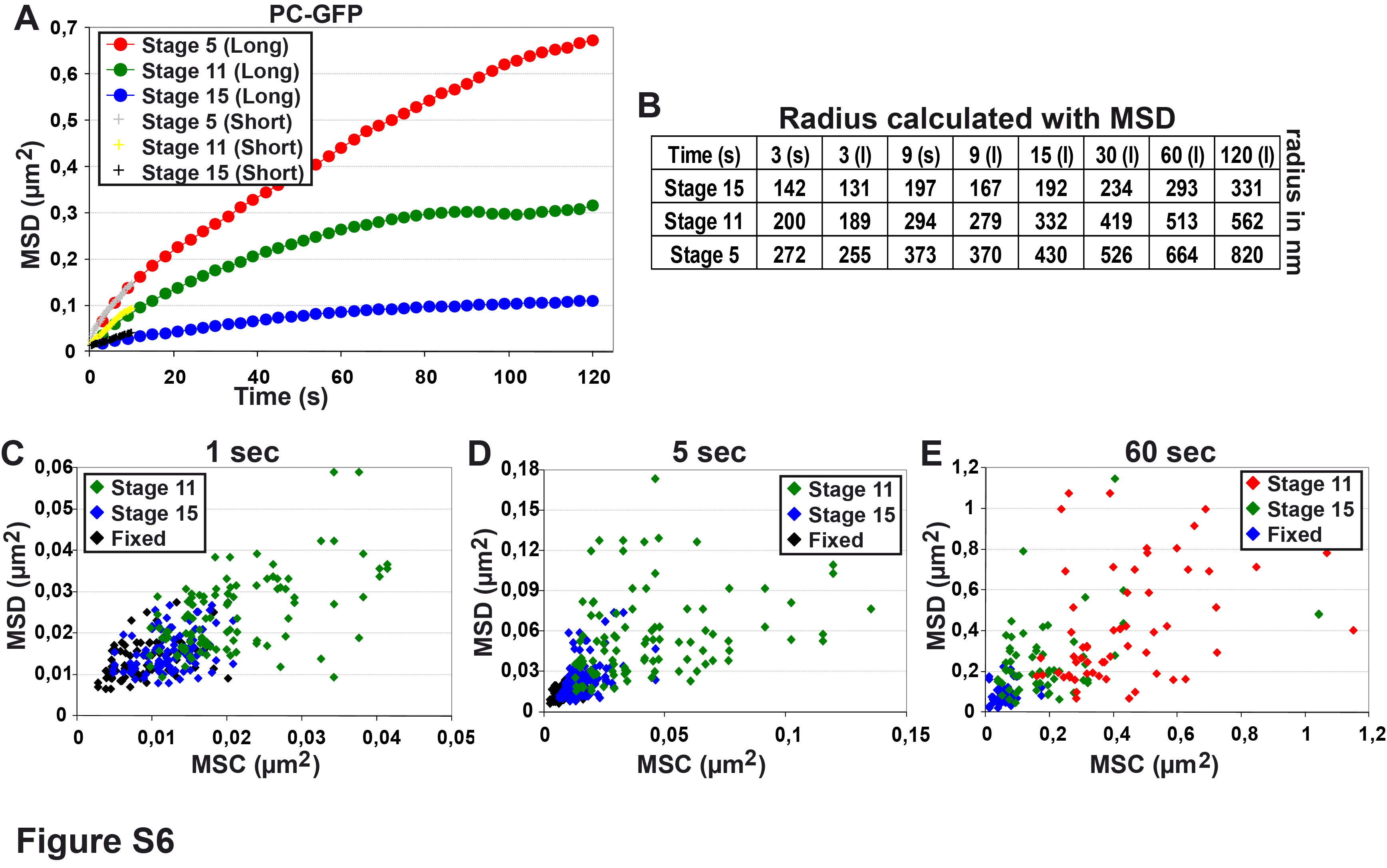

Supplement: Figure S6 — Complex motion of PC bodies during embryonic development. A: MSD curves characterizing the motion of PC bodies during embryogenesis. The results obtained with projections along the Z-axis of long 4D tracking fit with the data found with fast 2-D time-lapse experiments (compare the red, green and blue curves with their corresponding grey, yellow and black ones). B: Tables showing the average radius (in nm) of the volumes in which PC bodies move. C–E: Scatter-plots between MSD of each PC body and its corresponding MSC computed for motions of 1 s (E), 5 s (F) or 60 s (G). (TIF) [file pgen.1002465.s006.tif]

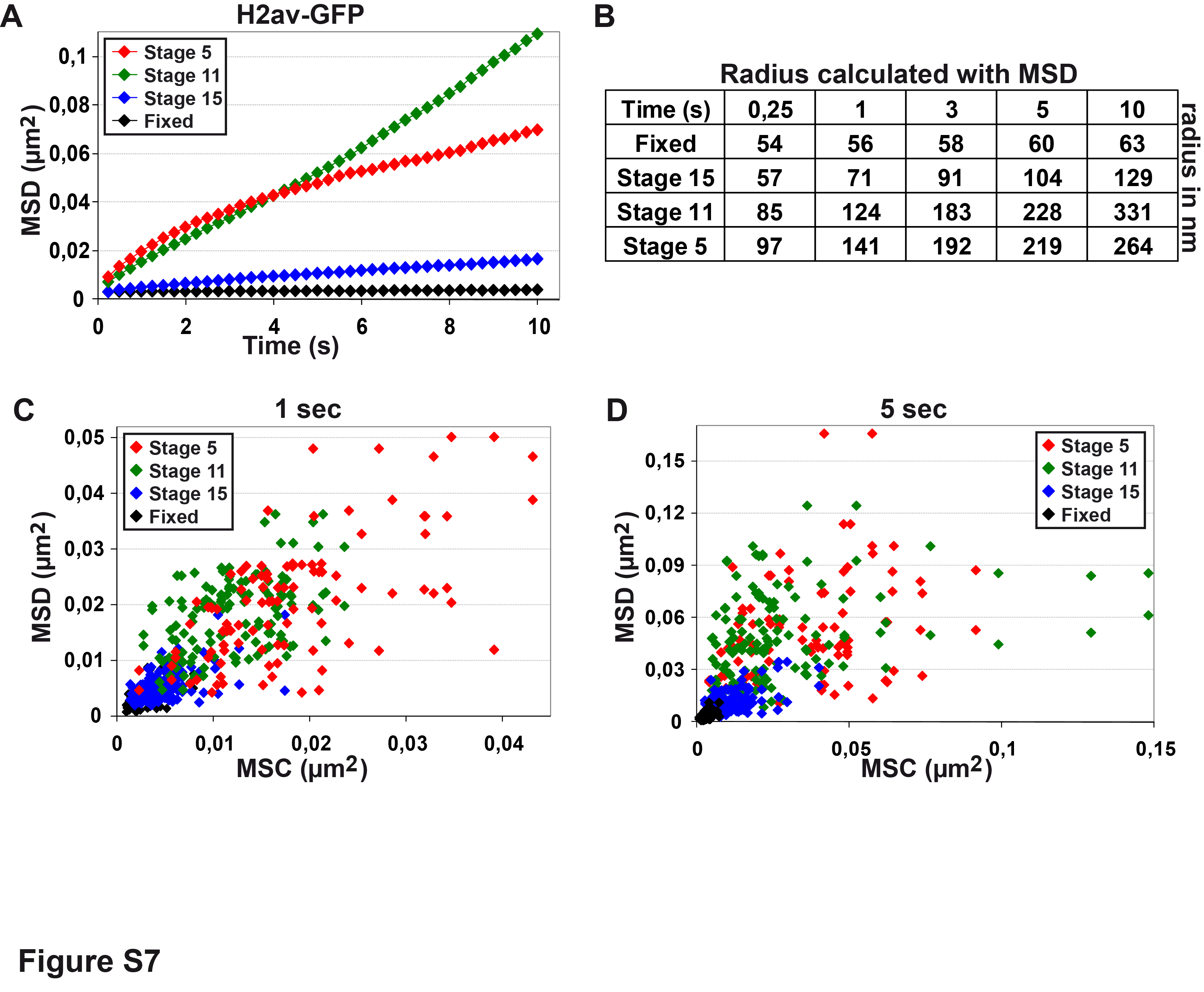

Supplement: Figure S7 — Complex motion of chromatin domains during embryonic development. A: MSD curves characterizing the motion of chromatin domains during embryogenesis. B: Tables showing the average radius (in nm) of the volumes in which chromatin domains move. C–D: Scatter-plots between the MSD of each chromatin domain and its corresponding MSC computed for motions of 1 s (E) or 5 s (F). (TIF) [file pgen.1002465.s007.tif]

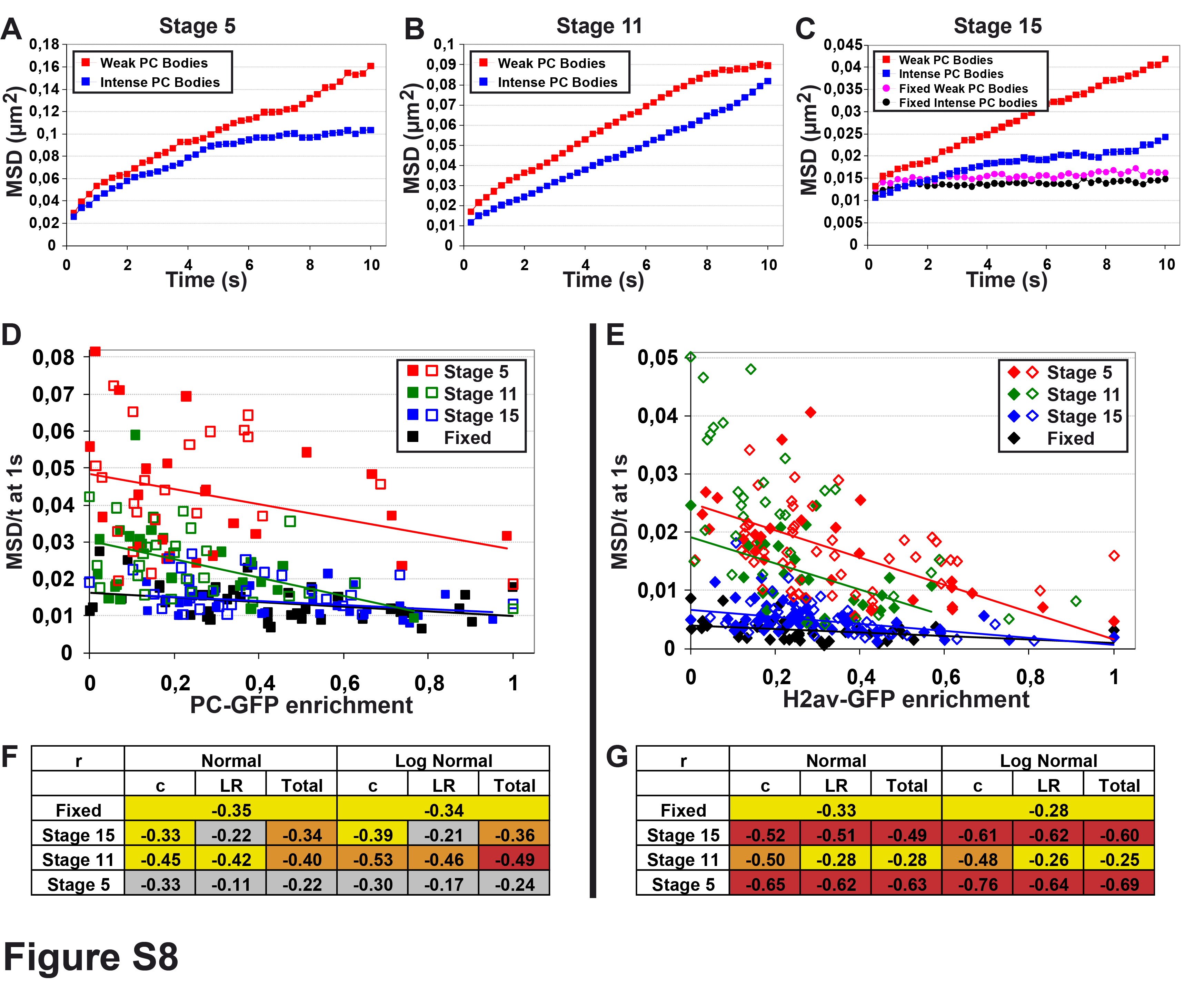

Supplement: Figure S8 — Effect of PC-GFP or H2Av-GFP enrichment on the motions of PC bodies or chromatin domains. A–C: MSD curves comparing the average motion between the most intense and the weakest PC body tracked within one nucleus at stages 5 (A), 11 (B) and 15 (C). D–E: Scatter-plots between the MSD/t reached after 1 s of PC bodies and chromatin domains and their respective enrichments in PC-GFP (D) or H2Av-GFP (E). Full squares and diamonds point tracks showing only constrained motion, whereas empty squares and diamonds correspond to tracks displaying both constrained and long-range motions. F–G: Tables presenting the coefficient of correlation (r) calculated between enrichments of PC-GFP (F) or H2Av-GFP (G) and the motions of PC bodies and chromatin domains (c = tracks only showing constrained motion; LR = tracks displaying both constrained and long-range motions; Total = c+LR). Colors depends on the p-value associated to the coefficient of correlation calculated for both normal and lognormal distributions of MSD/t (Red p<0.001; Orange p<0.01; yellow p<0.05 and grey p>0.05). (TIF) [file pgen.1002465.s008.tif]

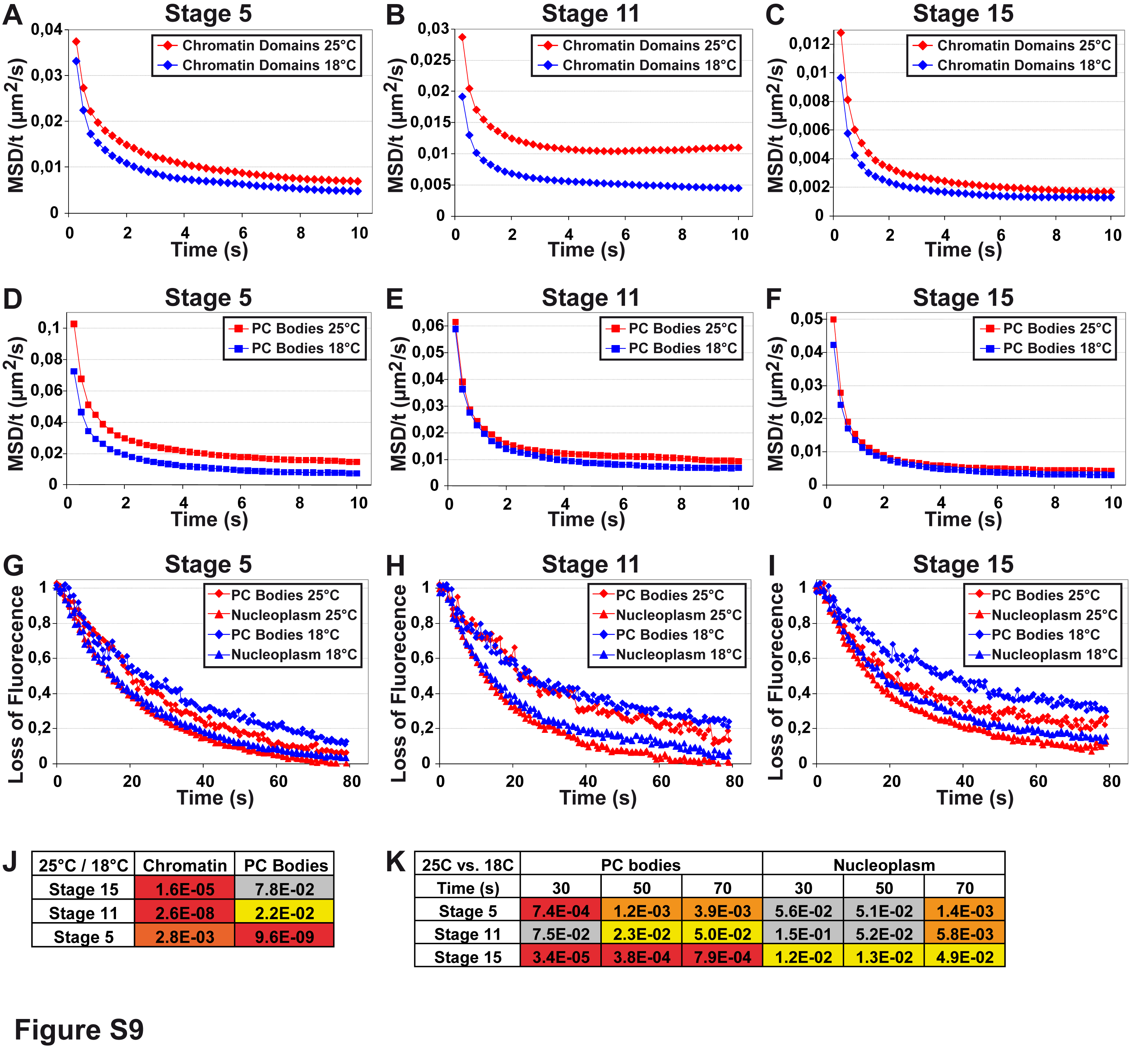

Supplement: Figure S9 — The motions of chromatin domains and PC bodies as well as the PC-GFP kinetics depend on temperature. A–F: Decrease of temperature slows down motions of chromatin domains and PC bodies. MSD/t curves quantifying the motions of chromatin domains (A–C) or PC bodies (D–F) at both 18°C and 25°C, in embryos at stages 5 (A and D), 11 (B and E) and 15 (C and F). G–I: Temperature affects kinetics of PC-GFP. Curves monitoring the loss of fluorescence occurring during FLIP experiments performed at both 18°C and 25°C in embryos at stages 5 (G), 11 (H) and 15 (I). J: Table of p-values comparing the MSD/t of chromatin domains and PC bodies reached after 5 s at 18°C with the ones calculated at 25°C. K: Table of p-values comparing the loss of fluorescence inside PC bodies and within the nucleoplasm at 18°C with the ones measured at 25°C. (J and K: red p<0.001; orange p<0.01; yellow p<0.05 and grey p>0.05). (TIF) [file pgen.1002465.s009.tif]

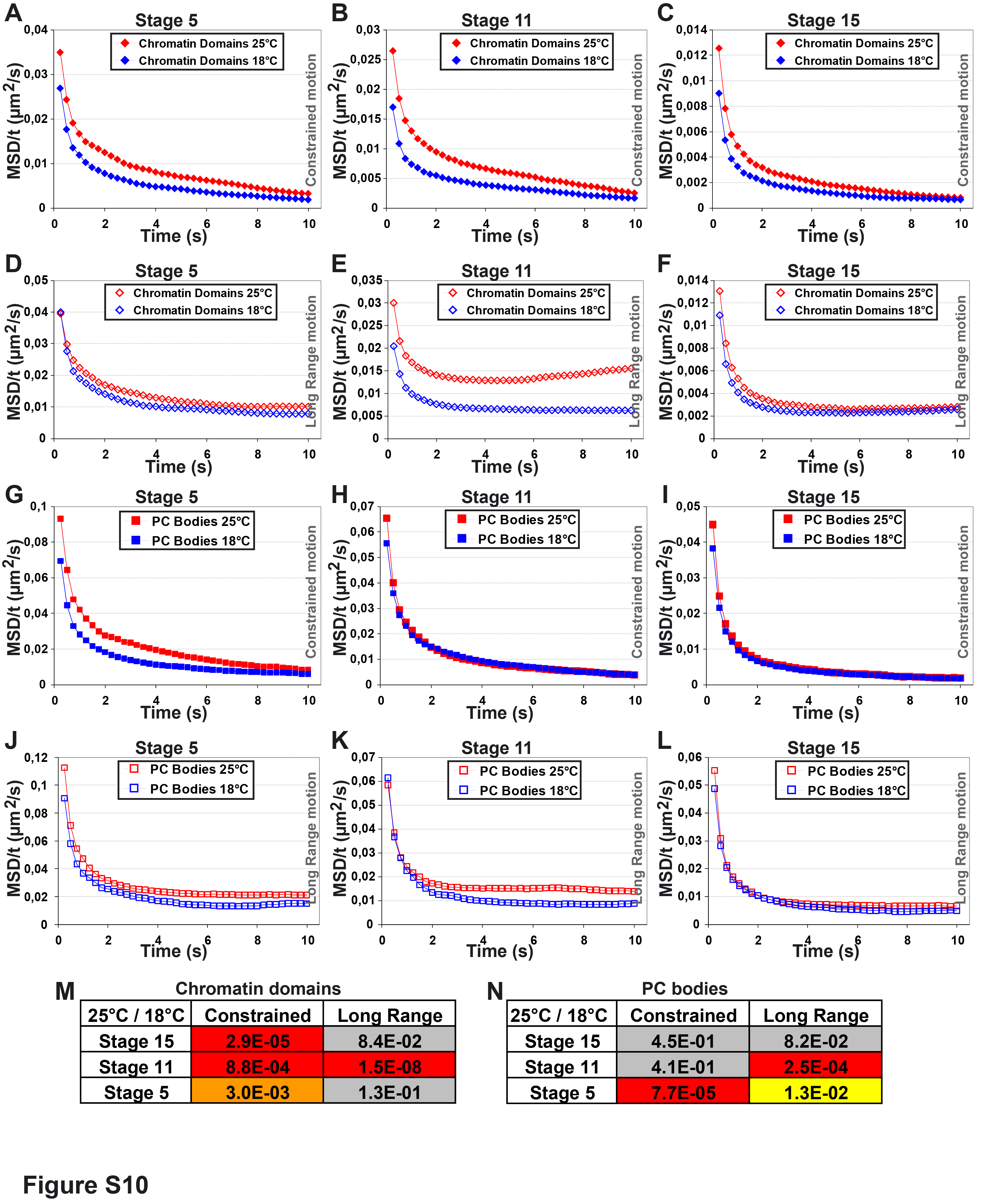

Supplement: Figure S10 — Effect of temperature on the motion of PC bodies and chromatin domains during embryogenesis. A–F: MSD/t curves quantifying constrained (A–C) and long-range (D–F) motions of chromatin domains at 18°C and 25°C, in embryos at stages 5 (A and D), 11 (B and E) and 15 (C and F). G–L: MSD/t curves quantifying constrained (G–I) and long-range (J–L) motions of PC bodies at 18°C and 25°C, in embryos at stages 5 (G and J), 11 (H and K) and 15 (I and L). M–N: Tables of p-values comparing the motions of chromatin domains (M) or PC bodies (N) measured at 18°C with the ones observed at 25°C. (TIF) [file pgen.1002465.s010.tif]
